# Phase-variable Type I methyltransferase M.NgoAV from *Neisseria gonorrhoeae* FA1090 regulates phasevarion expression and gonococcal phenotype

**DOI:** 10.3389/fmicb.2022.917639

**Published:** 2022-10-04

**Authors:** Monika Adamczyk-Poplawska, Pawel Bacal, Agnieszka Mrozek, Natalia Matczynska, Andrzej Piekarowicz, Agnieszka Kwiatek

**Affiliations:** ^1^Department of Molecular Virology, Institute of Microbiology, Faculty of Biology, University of Warsaw, Warsaw, Poland; ^2^Institute of Paleobiology, Polish Academy of Sciences, Warsaw, Poland

**Keywords:** restriction-modification, DNA methylation, *Neisseria gonorrhoeae*, phasevarion, pathogenicity, Type I MTase, NgoAV

## Abstract

The restriction-modification (RM) systems are compared to a primitive, innate, prokaryotic immune system, controlling the invasion by foreign DNA, composed of methyltransferase (MTase) and restriction endonuclease. The biological significance of RM systems extends beyond their defensive function, but the data on the regulatory role of Type I MTases are limited. We have previously characterized molecularly a non-canonical Type I RM system, NgoAV, with phase-variable specificity, encoded by *Neisseria gonorrhoeae* FA1090. In the current work, we have investigated the impact of methyltransferase NgoAV (M.NgoAV) activity on gonococcal phenotype and on epigenetic control of gene expression. For this purpose, we have constructed and studied genetic variants (concerning activity and specificity) within M.NgoAV locus. Deletion of M.NgoAV or switch of its specificity had an impact on phenotype of *N. gonorrhoeae*. Biofilm formation and planktonic growth, the resistance to antibiotics, which target bacterial peptidoglycan or other antimicrobials, and invasion of human epithelial host cells were affected. The expression of genes was deregulated in gonococcal cells with knockout M.NgoAV gene and the variant with new specificity. For the first time, the existence of a phasevarion (phase-variable regulon), directed by phase-variable Type I MTase, is demonstrated.

## Introduction

The restriction-modification systems (RM systems) are found in prokaryotic organisms and are compared to a primitive innate immune system, composed of methyltransferase (MTase) and restriction endonuclease (RE). The main function of DNA MTases of the RM systems is the labeling of bacterial own genetic material to protect it from degradation by the active REs. In turn, REs control the invasion of bacteria by foreign DNA (phages or plasmids) (Vasu and Nagaraja, 2013; Seib et al., 2020; Seong et al., 2021). However, it is currently apparent that the biological significance of RM systems extends well beyond this defensive function (Vasu and Nagaraja, 2013).

The methylation of DNA is also an epigenetic marker, as it enhances the information contained in the DNA sequence. In prokaryotes, it has been shown that DNA methylation is involved in the regulation of gene expression and initiation of DNA replication, as well as in the postreplicative DNA repair and virulence (Seib et al., 2020; Seong et al., 2021). However, such an epigenetic role has been known for a long time only for orphan MTases, including the Dam MTase (Low et al., 2001). Based on their structure and genetic organization, as well as the differences in the cleaving process and the cofactor requirements, the RM systems are classified into four types (I–IV) (Roberts et al., 2015). Several research groups have demonstrated that the MTases belonging to Type III RM systems may also affect the global gene transcription in different bacterial species such as *Neisseria gonorrhoeae*, *Neisseria meningitidis*, *Haemophilus influenzae*, *Helicobacter pylori*, or *Moraxella catarrhalis* (Srikhanta et al., 2005, 2009, 2010, 2011; Kwiatek et al., 2015; Blakeway et al., 2019). The variation of the level of gene expression, which may lead to the changes in the phenotype of bacteria, might be related to the expression of phase-variable *mod* genes, encoding the Type III MTases and to the differences in methylation patterns of genomic DNA (Srikhanta et al., 2010, 2011).

The phase-variation (PV) of Type III MTases consists in general in switching ON and OFF, the methylation activity encoded by the *mod* gene. Srikhanta et al. (2005) proposed the existence of phasevarion (phase-variable regulon), i.e., a group of genes, commonly regulated by phase-variable Type III MTases. Since such Type III MTase-directed phasevarions were discovered in different bacteria (Srikhanta et al., 2011; Kwiatek et al., 2015), the existence of two phasevarions regulated by phase-variable Type III MTases was demonstrated in *N. gonorrhoeae* FA1090 strain (Srikhanta et al., 2005; Kwiatek et al., 2015). PV of these two groups of genes modulates the gonococcal FA1090 strain’s viability, pathogenicity toward human cells, and the ability to form biofilms (Srikhanta et al., 2005, 2009; Kwiatek et al., 2015).

In contrast, the data on the role of MTases belonging to Type I RM systems in the regulation of gene expression are very limited (De Ste Croix et al., 2017; Seib et al., 2020).

Canonical Type I RM systems are encoded by three genes: *hsdS*, *hsdM*, and *hsdR*. The complexes of HsdM and HsdS subunits form a catalytically active MTase and also the combination of HsdM, HsdS, and HsdR subunits–an enzyme with both endonucleolytic and modification activities. The specificity of the Type I RM system is determined by the HsdS subunit (Murray, 2000).

We have previously characterized Type I RM system, NgoAV encoded by *N. gonorrhoeae* FA1090. This system is atypically encoded by *hsdM*, *hsdS1*, *hsdS2*, and *hsdR* genes (Adamczyk-Poplawska et al., 2011). The specificity subunit, HsdSNgoAV, the product of the *hsdS1* gene, is a naturally truncated form of an archetypal specificity HsdS subunit (208 N-terminal amino acids instead of 410). The *hsdS2* gene does not encode an active protein. The existence of the truncated *hsdS1* gene is caused by a frameshift associated with the presence of a polyG tract at the 3′ end of *hsdS1* gene. We have also shown that the *hsdS1* gene, encoding the specificity subunit of the Type I NgoAV RM system, is phase-variable and that PV is associated with the changes in the number of guanines in the homonucleotide polyG tract. PV consisting of deletion of one guanine within *hsdS1* gene results in fusion of *hsdS1* and *hsdS2* genes and the shift in the specificity of RM system from GCAN_8_TGC (NgoAV specificity) sequence to GCAN_7_STCA (S=G or C) (NgoAVΔT specificity). The addition of one G does not seem to affect the M.NgoAV (methyltransferase from NgoAV RM system) activity *in vitro* (Adamczyk-Poplawska et al., 2011).

We hypothesized that Type I MTases may regulate the expression of a group of genes, similar to Type III MTases. Moreover, we postulated that the activity of the phase-variable Type I M.NgoAV affects the expression of a group of gonococcal genes belonging to “Type I MTase dependent regulon”–the Type I MTase-directed phasevarion. To confirm our hypothesis, we have constructed different variants within the locus encoding M.NgoAV. The impact of modulation of the phasevarion has been studied on bacterial phenotype: the ability to fit into different environmental conditions and on bacterial pathogenicity and virulence, including interactions with host cells. Then, we determined the group of genes belonging to the phasevarion directed by M.NgoAV, by comparing the global expression level of gonococcal genes in the presence and absence of M.NgoAV activity.

## Materials and methods

### Bacterial strains growth conditions

*Escherichia coli* strain Top10 [F’[lacIq Tn10(Tetr)] *mcrA* Δ(*mrr-hsdRMS-mcrBC*) *φ80lacZ*Δ*M15* Δ*lacX74* nupG recA1 araD139 Δ(*ara-leu*)7697 *galE15 galK16 rpsL*(Str*^r^*) *endA1* AAA-] was grown in Luria-Bertani (LB) broth (Difco, United States) or on LB agar at 37°C. The following antibiotics were used for *E. coli* when needed, kanamycin (30 μg/ml) or chloramphenicol (34 μg/ml) or ampicillin (100 μg/ml).

*Neisseria gonorrhoeae* FA1090 and variant strains were grown on GC agar base (Difco, United States) supplemented with 1% Kellogg’s supplement and 1% hemoglobin at 37°C in 5% CO_2_ or in GC broth with 1% Kellogg’s supplement and 0.043% NaHCO_3_ (Dillard, 2011). When needed, kanamycin (30 μg/ml) or chloramphenicol (0.75 μg/ml) was added to GC. Before each experiment, inoculum of a predominantly Pili^+^ Opa^+^ frozen stock of gonococci was spread and cultivated for 24 h on GC agar base without hemoglobin to evaluate gonococcal colony morphologies under a stereo dissecting microscope following the principles described by Dillard (2011). Next, Pili^+^ and opaque phenotype colonies were picked, streaked on GC agar base supplemented with hemoglobin, and cultivated for the next 24 h.

### Construction of *Neisseria gonorrhoeae* variant strains: the knockout mutant in NgoAV RM system (*ngoAVhsdS1::cm*) called NgoΔAV, mutant with new specificity (*ngoAVhsdS*Δ*T*) called NgoAVΔT, and the complementation mutant (*ngoAVigatrpb::hsdS1*) called compNgoAV

#### Construction of *Neisseria gonorrhoeae* NgoΔAV (*ngoAVhsdS1::cm*)

To construct the NgoAV knockout mutant, we replaced the *hsdS1* gene on the FA1090 chromosome with an allele disrupted by chloramphenicol cassette (*cm*). Plasmid pKRP10 (Dziewit et al., 2011), donor of *cm* cassette, was cut with SmaI. Purified from agarose, 800-bp DNA fragment was then cloned into pMS2 (encoding M. NgoAV) (Adamczyk-Poplawska et al., 2011), linearized with EcoRV. Obtained plasmid pMS2::cm was then sequenced to confirm the insertion of cm into the *hsdS1* gene and used to transform piliated, i.e., competent *N. gonorrhoeae* FA1090 cells. Linearization of pMS2::cm with NdeI forced the replacement of the wild-type (wt) *hsdS1* gene by the *hsdS1::cm* allele in the genome of FA1090, by the occurrence of double cross-over. *N. gonorrhoeae* colonies, selected on GC plates, containing chloramphenicol, were used for colony PCR with primers hsdSXylR and hsdSXylL (refer to Supplementary Table 4 for primer sequences) using PfuUltra II Fusion HS DNA polymerase (Agilent Technologies, United States), according to the manufacturer’s protocol. From colonies with wt *hsdS1* gene, a fragment of 1,759 bp would be obtained, whereas a fragment of 2,700 bp was obtained when the *hsdS1* gene was interrupted by cm cassette. The selected mutant strain, lacking wt M.NgoAV (knockout mutants), was called NgoΔAV (*ngoAVhsdS1::cm*).

#### Construction of *Neisseria gonorrhoeae* with the complementation of deleted *hsdS1* gene: mutant compNgoAV (*ngoAVigatrpb::hsdS1*)

First, we obtained plasmid pkompNgoAV in which the *hsdS1* gene was cloned into intergenic region *iga-trpb* on vector pMPMigatrpBopaKM (pMPMA4Ω vector with cloned intergenic region *iga-trpb* and *km* cassette) (Kwiatek et al., 2015). For this, the *hsdS1* gene was amplified from purified chromosomal DNA of *N. gonorrhoeae* FA1090 (Gene Bank: AE004969, ATCC 700825) by PCR with NhehsdS and SmahsdS primers (Supplementary Table 4) using PfuUltra II Fusion HS DNA polymerase. The resulting fragment of 675 bp corresponding to the wt *hsdS1* gene was ligated with the PCR fragment (6,578 bp) obtained by amplification using the pMPMigatrpBopaKM vector as template and primers Smatrpb and Nheiga (Supplementary Table 4). In the obtained construct, the *hsdS1* gene is under the control of a strong constitutive P_*opa*_ promoter as previously described (Ramsey et al., 2012; Kwiatek et al., 2015).

Next, *N. gonorrhoeae* NgoΔAV strain was transformed with plasmid pkompNgoAV linearized with Kpn2I. *N. gonorrhoeae* cells, in which the interrupted *hsdS1* gene was complemented with the wt *hsdS1* gene, inserted between *iga* and *trpb* genes, were selected on GC plates containing both chloramphenicol and kanamycin.

Colonies were verified by colony PCR (with primers NhehsdS and SmahsdS). For further analysis, we selected colonies in which we obtained 2 PCR products (675 bp corresponding to wt copy of *hsdS1* gene from compNgoAV and 1586 bp corresponding to *hsdS1* locus interrupted by *cm* cassette). For further confirmation of insertion of *hsdS1* locus into interregion between *iga* and *trpb* genes, a PCR with primers Nheiga and SmatrpB was performed. Clones in which we have obtained PCR product of 2,981 bp have been selected for further investigations (in case of the lack of proper insertion, we would obtain PCR product of 1,134 bp, corresponding to wt *iga-trpb* locus). Appropriated PCR products were sequenced for the confirmation of adequate sequences. The resulting recombinant strain (*ngoAVigatrpb::hsdS1*) was called *N. gonorrhoeae* compNgoAV (complementation mutant).

#### Construction of *Neisseria gonorrhoeae* with new specificity: fusion of *hsdS1* and *hsdS2* genes: mutant NgoAVΔT (*ngoAVhsdS*Δ*T*)

First, plasmid pSR encoding *hsdS* (fused in frame *hsdS1* and *hsdS2* genes) and *hsdR* genes was obtained by the deletion of *hsdM* gene from previously described plasmid pNo12ΔT (Adamczyk-Poplawska et al., 2011). For this, pNo12ΔT was digested by EcoRI and self-ligated. Then, the *km* cassette was inserted between *hsdS* and *hsdR* genes on pSR. For this, PCR fragment obtained by ExSite PCR with primers MAP16a and MAP17a and digested by PstI was ligated with PstI-digested km cassette from plasmid pDIY-km (Dziewit et al., 2011). Obtained pSR-km, linearized by NdeI, was used to transform piliated *N. gonorrhoeae* FA1090 and transformed cells were selected on GC containing kanamycin.

For the confirmation of insertion of fused *hsdS* gene, we used previously described system for the detection of PV of M.NgoAV (Adamczyk-Poplawska et al., 2011). In this system, we used pMPMlacZ vector in which we cloned PCR product obtained with primers HsdSfor and HsdSrev (Supplementary Table 4) into EcoRI and NcoI sites. Plasmid pMPMlacZ was constructed in such way that α-complementation is possible only if the *hsdS1* gene is fused in-frame to *hsdS2*. The blue color of transformed *E. coli* indicated the proper exchange of alleles (Adamczyk-Poplawska et al., 2011). For further studies, we selected kanamycin-resistant *N. gonorrhoeae* cells that allow obtaining blue cells after cloning of PCR fragment in pMPMlacZ. Moreover, the proper fusion of *hsdS1* and *hsdS2* genes, due to the replacement of alleles, was confirmed by sequencing with primer HsdSfor.

#### *Ex vivo* verification of constructed *Neisseria gonorrhoeae* NgoAVΔT (*ngoAVhsdS*Δ*T*) mutant specificity

After the confirmation of proper exchange of wt and fused alleles, we verified that constructed NgoAVΔT mutant actually encodes active MTase with M.NgoAVΔT specificity.

For this purpose, from kanamycin-resistant *N. gonorrhoeae* colonies, in which we confirmed the insertion of the fused *hsdS* gene as described above, we amplified (with primers MAP20 and MAP21) the *hsdM* and *hsdS* genes (encoding M. NgoAVΔT) and cloned the obtained PCR product into PstI and HindIII sites of pUC19 vector. The proper cloning was verified by sequencing. Then, isolated, recombinant plasmid DNA was submitted to HincII digestion, and the products of digestion separated on 0.7% agarose gel (Adamczyk-Poplawska et al., 2011).

Another test involved the restriction of λ phage by NgoAV or NgoAVΔT RM systems cloned in *E. coli* cells (Adamczyk-Poplawska et al., 2011). The evaluation of the methylation pattern of λ phages was performed by double-agar test (Sambrook, 2001).

Construction of all three mutants, NgoΔAV, NgoAVΔT, and compNgoAV, was also confirmed by Southern Blot. Chromosomal DNAs were isolated from constructed variants and 1.0 μg digested with MluI. Obtained fragments were separated on agarose gels (0.7%) at 130 V for 2 h and Southern alkali transfer, followed by hybridization (Sambrook, 2001). We used a non-radioactively labeled km or cm cassette (DIG-High Prime, Roche) as the probe, to confirm proper insertions of antibiotic cassettes.

### Field emission scanning electron microscopy

In total, three constructed mutants and FA1090 wt strains were grown on GC plates for 24 h. Cells were then harvested and cell suspensions in GC broth were made to OD_600_ = 0.05 (10^7^ cells/ml). Gonococcal suspensions were cultivated in GC broth on cover glasses placed in Petri dishes for 24 h at 37°C in 5% CO_2_. Then, formed biofilms were soaked in 3% glutaraldehyde in 0.1 M cacodylate buffer (pH 7.3) for 24 h. Next, samples were rinsed five times with cacodylate buffer. Samples were then dehydrated by dipping in 96% ethanol for 6 h and air-dried. After plasma coating with gold-palladium (circa 2–4 nm thick), biofilms were analyzed with the field emission scanning electron microscope (FE SEM) (MERLIN Carl Zeiss Germany) at a 2–5 kV range accelerating voltage. The morphology of samples was investigated at various magnification ranges up to nanometer image resolution, using secondary electron detectors-conventional side (ET) and in-lens. The measurement of bacterial cells size was performed in arbitrary units using ImageJ (Schneider et al., 2012). We measured the size of at least 20 cells/sample.

### Microtiter-plate adherence assay

Microtiter-plate adherence assays using crystal violet were performed as described (Kwiatek et al., 2015). Briefly, *N. gonorrhoeae* wt and mutant strains were harvested from GC agar after 24 h of growth and cell suspensions with OD_600_ = 0.05 were prepared in GC broth with Kellogg’s and 5 mM MgSO_4_. After plating into 96-well microtiter plates, gonococci were cultivated for 4, 6, or 24 h at 37°C in 5% CO_2_. Then, the OD_600_ was measured. Next, the supernatant was aspirated, and each well was extensively washed 3 times with sterile phosphate-buffered saline (PBS) to remove all non-adherent bacteria. The attached bacteria were then stained with 0.5% crystal violet for 15 min. After extensive washing with tap water, the dye bound to biofilm-forming cells was solubilized with ethanol, and OD_570_ was measured. Approximately twelve technical measurements were taken for each mutant. Experiments were carried out three times and results were averaged.

### RNA isolation

RNA isolation from wt and studied mutants was performed as described previously (Kwiatek et al., 2014, 2015). Total RNAs were isolated with High-Pure RNA Isolation Kit (Roche Life Science, Switzerland) and DNA-free, DNase Treatment and Removal (Ambion, United States), according to the manufacturer’s recommendations from gonococci growing on one GC agar plate (no pooled samples) for 24 h. From one plate, approximately 20–30 μg of total bacterial RNA was purified. RNA quality was evaluated with 2100 Bioanalyzer (Agilent Technologies, United States). Only samples with RIN >8.5 were chosen for microarrays. In total, four wt samples and four NgoΔAV samples were chosen for further analysis.

### Microarray experiments

Two-color RNA microarray analysis was performed using the Agilent-034141 array as described (Kwiatek et al., 2014, 2015). The Cy5 dye was used to label the cRNA of the NgoΔAV (*ngoAVhsdS1::cm*) strain and the Cy3 dye to label the cRNA of wt *N. gonorrhoeae* FA1090 strain. In total, four wt and four NgoΔAV mutant biological replicates were analyzed for microarray statistical analysis.

### Microarray analysis

Data files from the Agilent G2565CA Microarray Scanner System were analyzed using the GeneSpring (Agilent Technologies, United States, version 12.5) as described previously (Kwiatek et al., 2014, 2015). Variations were presented as the ratio of gene expression of wt gonococcal strain over-expression of the same gene for the NgoAV knockout mutant (strain NgoΔAV: *ngoAVhsdS1::cm*). Genes with different expressions at least 2.0-fold and a *p*-value < 0.05 were further analyzed. Microarray data have been deposited in the National Center for Biotechnology Information (NCBI) and are accessible through Gene Expression Omnibus series under the accession number GSE71703 (Edgar et al., 2002).

### Real-time qRT-pCR

The Maxima First-Strand cDNA Synthesis Kit (Thermo Scientific) was used to reverse-transcribe the 10 μg of total RNAs. Real-time PCR (HOT FIREPol^®^ EvaGreen^®^ qPCR Mix Plus, Solis BioDyne, Estonia) was carried out on the Applied Biosystems^®^ StepOne™ Real-Time PCR Systems (Life Technologies, United States). For each gene of interest, we used HPLC-purified oligonucleotide primers from Sigma-Aldrich (refer to Supplementary Table 4).

The comparative threshold cycle (ΔΔCt) method was used for the relative quantification of gene transcription. The 16S rRNA gene was used to normalize the relative amount of target cDNA as an internal reference (Kwiatek et al., 2014, 2015). Data represent averages obtained by measurement of three independent biological samples.

### Antibiotic resistance and toxicity tests

Kirby–Bauer disk diffusion susceptibility test protocol (Hudzicki, 2009) was adapted to test the toxicity of different chemical compounds for gonococci. Briefly, overnight cultures of *N. gonorrhoeae* were collected in 3 ml of sterile saline. OD was adjusted to 0.5 in the MacFarland scale and cells were plated (by streaking the swab three times over the entire agar surface) on GC with Kellogg and hemoglobin. Disks with antibiotics or other substances were then applied and plates incubated for 24 h at 37°C and 5% CO_2_. Then, the diameter of the inhibition zone of growth was measured in cm. Oxoid antibiotic susceptibility disks were used to test recombinant mutants and wt *N. gonorrhoeae* strain sensitivity to antibiotics. We used disks with miniscule 30 μg/ml, imipenem 10 μg/ml, bacitracin 10 μg/ml, azithromycin 15 μg/ml, gentamycin 30 μg/ml, and polymyxin B 300 μg/ml (Oxoid, United Kingdom).

We also used Whatman filter paper disks on which we applied 5 μl of 30% H_2_O_2_, Triton-X100, or 10% SDS. Tests were carried out at least three times and the results were averaged. The significance of results was determined as described in Section “Statistical analysis.”

### Adhesion and invasion assay of *Neisseria gonorrhoeae* variants to human cells

The human adenocarcinoma endometrial cells Hec-1-B (ATCC, HTB113) were cultivated in DMEM (Sigma-Aldrich) supplemented with 10% fetal bovine serum (Biowest, France), 2 mM glutamine (Biowest, France), and 1 mM sodium pyruvate (Sigma-Aldrich). Cells were incubated at 37°C in 5% CO_2_ and passaged every 5 days. Hec-1-B cells were seeded in 24-well Cellstar plates (Greiner Bio-One GmbH, Germany) at a density of 10^5^ cells/well and incubated at 37°C in 5% CO_2_. Bacteria were added when human cells formed a monolayer (in general after 5 days of culture). Adhesion and invasion assays were performed as described previously (Kwiatek et al., 2014, 2015).

The adhesion index was the quotient of dividing the human cell-associated bacteria (measured as colony-forming units–CFU) by the total number of bacteria (total CFU). The invasion index was calculated by dividing the number of intracellular bacteria by the adhesion index as described previously (Kwiatek et al., 2014, 2015). Tests were carried out at least three times and the results were averaged. The significance of results was determined as described in Section “Statistical analysis.”

### Statistical analysis

The significance of the results was determined by performing first the Shapiro–Wilk test to test the normality of data distribution. Then, we tested the null hypothesis that groups had the same median using the non-parametric Kruskal–Wallis test. When the null hypothesis was rejected, we performed the Bonferroni adjustment to reduce the instance of a false positive significance.

## Results

### *In silico* analysis of *ngoAV*-like locus in sequenced gonococcal genomes

The nucleotide sequence encoding the *ngoAV* locus (399256-400478 nt) from *N. gonorrhoeae* FA1090 (AE004969.1) was compared to other sequenced gonococcal genomes available in NCBI database. BlastN comparison was enhanced by careful analysis of the sequences that have more than 97% identity and 100% cover to the studied sequence. We have grouped the selected strains according to the number of guanines (G) within the polyG tract present at the end of *hsdS1* gene, the possibility to encode a functional HsdS1 subunit (MTase with NgoAV specificity) or fused HsdS subunit (MTase with NgoAVΔT specificity). We also attempted to correlate the number of G with the isolation site of gonococci.

**FIGURE 1:**
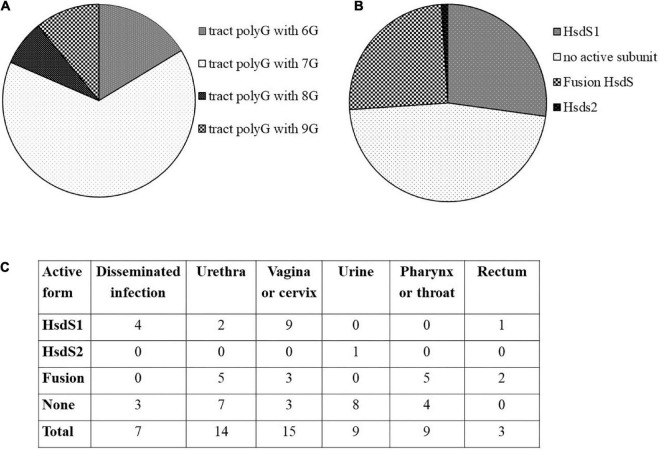
*In silico* analysis of *ngoAV* locus present in sequenced genomes of gonococci from NCBI database. **(A)** Distribution of the number of guanines (G) in the polyG tract. **(B)** Distribution of probable active and non-active forms of MTase forming subunits. **(C)** Number of strains according to their isolation site and the probable form of NgoAV-like MTase.

Our investigation revealed 92 gonococcal strains containing *ngoAV*-like locus. In total, sixty of them (>65% of analyzed sequences) have a polytract of 7G (as *N. gonorrhoeae* FA1090 strain). In total, fifteen strains have 6G-containing polyG tract, 7 have 8G, and 10 have 9G within the polyG tract. Detailed investigation demonstrated that 25 strains (27%) encode a probably active MTase with NgoAV specificity (functional HsdS1 subunit and M.NgoAV specificity), 23 (25%) encode possibly a fused subunit (NgoAVΔT specificity), and 43 (47%) presumably did not encode any active protein M.NgoAV-like, because of additional mutations within the *hsdS1* gene and the frameshift due to the presence of 7G within polyG tract. In total, one strain (NJ189125) encodes probably an active MTase encoded by an elongated at 5′ end *hsdS2* gene (possibly a functional HsdS2 subunit). *N. gonorrhoeae* MS11 strain may encode two functional HsdS1 subunits. The attempts to find a correlation between the encoded form of *ngoAV* locus and the isolation source of bacteria are biased by the fact that in the majority of cases, the information about the site of isolation of bacteria is missing. However, some forms of *ngoAV* locus seem to be found more often in the case of male (specificity NgoAVΔ like determined by fusion form or no active Type I MTase) vs. female (the specificity determined by HsdS1) infection by gonococci. As it can be seen in Figure 1C, in case of isolation of clinical samples from men (i.e., from urethra), the *ngoAV* locus encoded the fusion M.NgoAVΔT form of MTase (36% of cases) or is predicted as not encoding any active NgoAV-like MTase (50% of cases). Isolated from men, only 14% of *ngoAV* locus would encode the truncated M.NgoAV form. In case of isolation of gonococci from women (vagina or cervix) in 60% of cases, the truncated form of HsdS subunit (M.NgoAV) was observed according to G number in polyG tract. All the results are presented in Figure 1 and Supplementary Table 1.

### *Neisseria gonorrhoeae ngoAVhsdS*Δ*T* mutant (called NgoAVΔT) encodes active methyltransferase with M.NgoAVΔT specificity

A mutant strain of *N. gonorrhoeae* encoding M.NgoAVΔT MTase was obtained by homologous recombination between wt copy of *hsdS1* gene and fused *hsdS* gene from previously described pNo12ΔT (Adamczyk-Poplawska et al., 2011). The proper exchange of alleles, in *N. gonorrhoeae* cells, was proved as described in Section “Materials and methods.” Then, we confirmed, by two *ex vivo* tests, that constructed mutant actually produces an active MTase with M.NgoAVΔT specificity.

**FIGURE 2:**
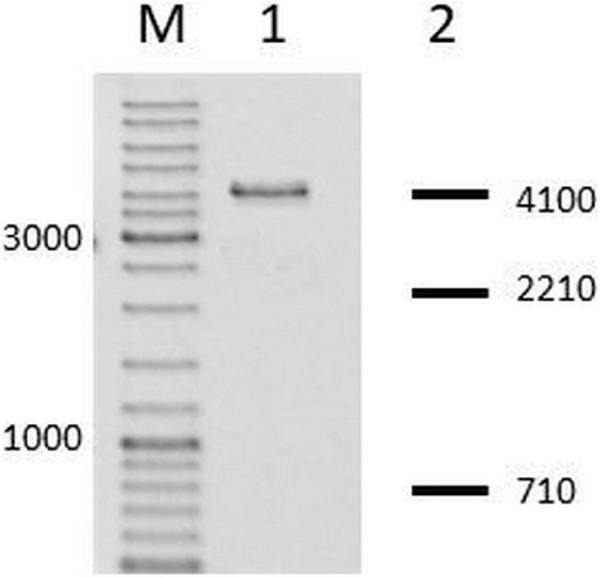
*Neisseria gonorrhoeae* cells with fused *hsdS1* and *hsdS2* genes produce active M.NgoAVΔT MTase. Lane M: GeneRuler DNA Ladder Mix: 10,000, 8,000, 6,000, 5,000, 4,000, 3,500, 3,000, 2,500, 2,000, 1,500, 1,200, 1,000, 900, 800, 700, 600, 500, 400, 300, 200, and 100 bp. Lane 1. Recombinant vector, uncleaved by HincII, due to methylation of overlapping sites by active M.NgoAVΔT MTase (Adamczyk-Poplawska et al., 2011). Lane 2: DNA fragments that would be obtained after digestion by HincII in case of lack of methylation by M.NgoAVΔT (numbers represent base pairs).

First, from *N. gonorrhoeae* colonies, in which we confirmed the insertion of the fused *hsdS* gene, we amplified genes encoding M.NgoAVΔT and cloned them into pUC19 vector. Then, the purified recombinant vectors were digested with HincII. HincII (recognized sequence: GTY↓RAC) is sensible to M.NgoAVΔT-specific methylation of DNA (GCAN_7_STCA) but not to M.NgoAV methylation (GCAN_8_TGC), as described previously (Adamczyk-Poplawska et al., 2011). In all cases, the digestion profile of vector DNA encoding M.NgoAVΔT confirmed the inhibition of HincII activity, indicating that *N. gonorrhoeae* cells with the fused *hsdS1* and *hsdS2* genes actually produce an active MTase with M.NgoAVΔT specificity instead of M.NgoAV. The example of the gel after electrophoresis of such sample is shown in Figure 2.

The second test involved the RM of λ phage in a double-agar test. Selected recombinant vectors resistant to HincII digestion and encoding putative M.NgoAVΔT were transformed to *E. coli* cells, which in turn were used for the propagation of λ phages. Collected phages λ? (“?” indicates the unknown modification pattern of viral DNA) were then replicated in *E. coli* (r^–^ m^–^) or *E. coli* encoding cloned wt NgoAV RM system or *E. coli* encoding cloned NgoAVΔT RM system (Adamczyk-Poplawska et al., 2011). As shown in Table 1, λ? phage is not restricted by *E. coli* (NgoAVΔT) cells and is restricted by *E. coli* (NgoAV) cells. These results indicated that cloned MTase from transformed *N. gonorrhoeae* is active and has the desired M.NgoAVΔT specificity.

**TABLE 1:** Determination of propagation ability of unmodified and modified λ phages.

	*λ*_0_ ^§^	*λ*_NgoAV_	*λ*_?_
*E. coli* (r^–^, m^–^)	4.98 (±0.3) × 10^8^	5.38 (±0.3) × 10^8^	8.9 (±0.4) × 10^8^
*E. coli* (NgoAV)	<10^5^	5.92 (±0.3) × 10^8^	<10^5^
*E. coli* (NgoAVΔT)	<10^5^	<10^5^	6.03 (±0.2) × 10^8^

^§^*λ*_0_ is unmodified bacteriophage propagated in the *E. coli* Top10 (r^–^ m^–^) strain. *λ*_NgoAV_ is modified *in vivo* by propagation in *E. coli* cells expressing NgoAV. *λ*_?_ is the phage which methylation specificity has to been determined. Values are given in pfu/ml. Data are means (±SD) of at least three independent experiments.

### Biofilm formation by *Neisseria gonorrhoeae* FA1090 is modulated by M.NgoAV methyltransferase

The effect of phase-variation of Type I M.NgoAV MTase on gonococcal phenotype was studied using wild-type (wt) strain of *N. gonorrhoeae* encoding NgoAV RM system and its derivatives: NgoΔAV (knockout mutant), NgoAVΔT (new specificity of Type I MTase), and compNgoAV (complementant strain).

**FIGURE 3:**
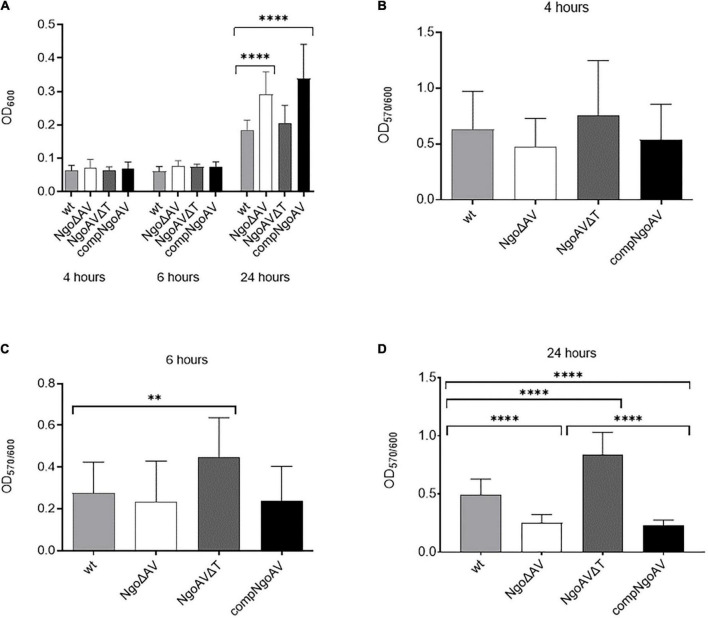
Growth and biofilm biomass of *N. gonorrhoeae* mutants within M.NgoAV locus compared to the wt strain. Measurements were performed by estimating bacterial density (OD_600_) and by staining with crystal violet (OD_570_) after a 4-, 6-, and 24-h growth. **(A)** Biofilm and planktonic cell growth were determined by absorbance OD_600_; **(B)** Biomass production: ratio of cells that form biofilm (OD_570_) vs. total grown cells (OD_600_) at 4 h. **(C)** Biomass production: ratio of cells that form biofilm (OD_570_) vs. total grown cells (OD_600_) at 6 h. **(D)** Biomass production: ratio of cells that form biofilm (OD_570_) vs. total grown cells (OD_600_) at 24 h. Asterisks represent statistically significant differences in comparison to the wt strain (*p* < 0.05). Data are means of at least three independent experiments (±SD).

First, gonococci under the study were examined for their growth and adherence abilities to polystyrene surfaces after 4, 6, and 24 h. At these time points, the overall density OD_600_, reflecting both planktonic and biofilm-engaged gonococcal cells, was determined. As shown in Figure 3A, the overall growth of mutant gonococci within NgoAV system was comparable to those obtained for the wt FA1090 strain at 4 and 6 h of culture (*p* > 0.05). After 24 h, the values of OD_600_ are similar for the wt and NgoAVΔT mutant strain (0.185 ± 0.076 and 0.205 ± 0.097, respectively; *p* > 0.05). The OD_600_ of knockout NgoΔAV strain and complementant strain were significantly larger than those of wt strain: OD_600_ for NgoΔAV strain was 0.292 ± 0.071 and for compNgoAV strain 0.338 ± 0.105. In both cases, the measured differences were statistically relevant, as compared to the value obtained for wt strain.

Subsequently, after 4, 6, and 24 h of growth, we measured the number of biofilm-forming cells by staining them with crystal violet (Figures 3B–D). No difference in biofilm formation was observed after 4 h of growth (Figure 3B), but after 6 h, the NgoAVΔT mutant strain formed a 1.6-fold larger biofilm biomass than the wt strain (OD_570/600_ = 0.447 ± 0.189 and 0.275 ± 0.149 for NgoAVΔT and wt strains, respectively; *p* < 0.05) as determined by comparing the biofilm biomass to the total amount of cells. The OD_570/600_ ratio observed for the strain with knockout M.NgoAV (OD_570/600_ = 0.235 ± 0.194) was similar to that obtained for wt gonococci after 6 h of cultivation (Figure 3C). Reintroduction of the wt copy of *hsdS1* gene (complementant) resulted in a similar number of cells forming biofilm at this time point (OD_570/600_ = 0.229 ± 0.165), as compared to the wt strain (*p* > 0.05). After 24 h, the proportion of NgoΔAV cells forming biofilm was smaller and the proportion of NgoAVΔT mutant cells was larger than proportion of wt cells forming biofilm (OD_570/600_ = 0.249 ± 0.073, OD_570/600_ = 0.842 ± 0.188, OD_570/600_ = 0.493 ± 0.135, respectively; *p* < 0.05). The introduction of the wt copy of *hsdS1* did not restore the biofilm formation at the level of wt strain at this time point: the biofilm density formed by compNgoAV mutant after 24 h of growth is at the level of knockout mutant and not wt strain (OD_570/600_ = 0.226 ± 0.049; Figure 3D).

**FIGURE 4:**
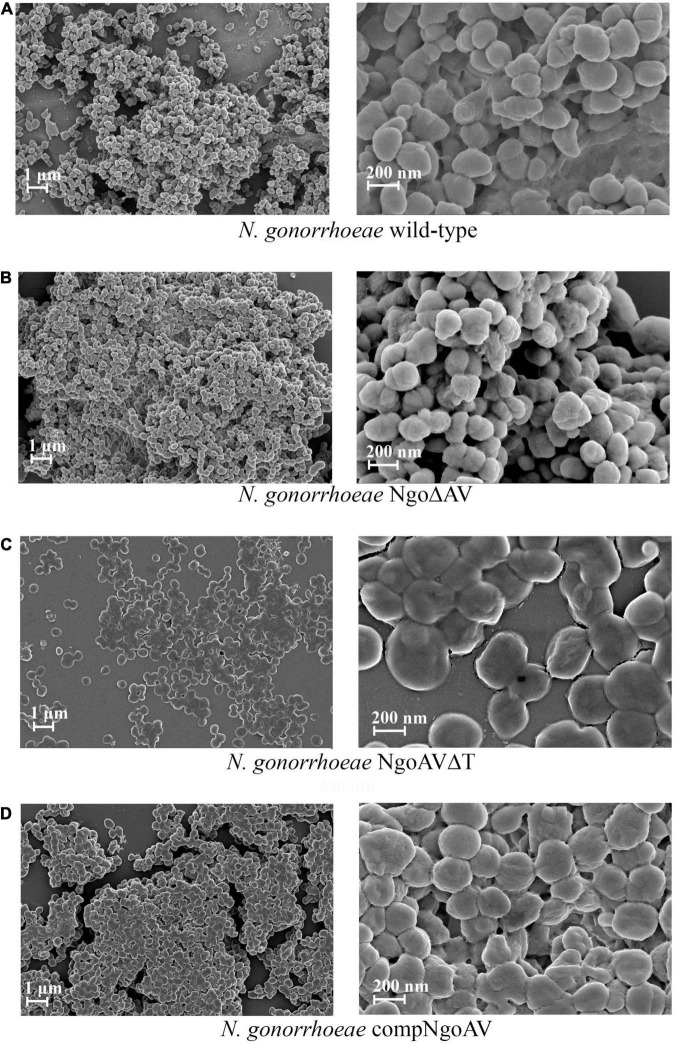
Biofilms produced by *N. gonorrhoeae* variants within M.NgoAV locus after 24 h of growth, visualized by Field Emission Scanning Electron Microscopy. **(A)** Biofilm formed by the wt FA1090 strain; **(B)** Biofilm formed by the *N. gonorrhoeae* NgoΔAV; **(C)** Biofilm formed by the *N. gonorrhoeae* NgoAVΔT mutant; **(D)** Biofilm formed by the *N. gonorrhoeae* compNgoAV strain. Experiments were triplicated and representative photographs are shown.

We have observed biofilms formed by gonococcal cells with varied expression of M.NgoAV under field emission scanning electron microscopy. Our analysis indicated the differences in biofilms formed by wt strain and mutants within M.NgoAV locus (Figure 4). Wild-type gonococci as well as cells with knockout M.NgoAV formed similar biofilm in structure and appearance without noticeable differences (Figures 4A,B). The biofilm formed by the mutant cells with changed specificity lacked depth: the biofilm appeared flat (Figure 4C). The phenotype of cells forming the biofilms also varied. Wild-type gonococcal, as well as NgoΔAV, cells forming biofilm had a spherical shape. In biofilm formed by the mutant with changed specificity, cells were flat and noticeably larger (Figure 4C). The complementation of M.NgoAV activity and specificity seemed to restore the biofilm appearance to similar, but not identical in shape to those formed by wt strain (Figure 4D).

**FIGURE 5:**
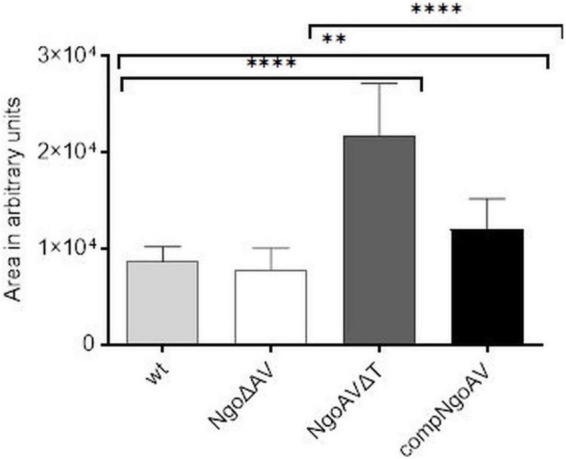
Sizes of individual *N. gonorrhoeae* cells from different variants within M.NgoAV locus, forming biofilm, seen under SEM and measured with ImageJ (in arbitrary units). Asterisks represent statistically significant differences in comparison to the wt strain and between NgoAVΔT and compNgoAV (*p* < 0.05). Data are means of area of at least twenty cells from three independent experiments (±SD).

We measured the area of gonococcal cells seen under FE SEM. For wt *N. gonorrhoeae* FA1090, we obtained a mean area of 8699.5 ± 1510.5 and a similar value was obtained for NgoΔAV mutant (7829.9 ± 2244.6; *p* > 0.05). The shift of the specificity of M.NgoAV (to M.NgoAVΔT) caused an increase in the area of cells by almost 3 times to 21701.9 ± 5479.3 (*p* < 0.05). Concerning the complementant mutant, the mean value of the measured area was 12008.6 ± 3182.9, which is significantly larger than the values obtained for wt strain (*p* < 0.05), but much smaller than the values obtained for NgoAVΔT mutant (*p* < 0.05). Results are given in arbitrary units as demonstrated in Figure 5.

### Invasion of *Neisseria gonorrhoeae* into human epithelial host cells is modulated by phase variable Type I M.NgoAV methyltransferase

**FIGURE 6:**
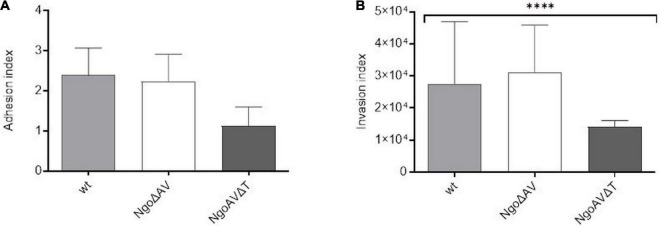
*Neisseria gonorrhoeae* adhesion and invasion indexes to human Hec-1-B cells. Human epithelial cells were infected for 4 h with the gonococcal mutants within NgoAV RM system and wt strain. **(A)** The adhesion index was calculated as a ratio between the numbers of bacteria that adhered to human cells to the total number of cells used for infection. **(B)** The invasion index was calculated by dividing the number of gentamicin resistant cells by the adhesion index. The asterisk represents the statistical difference in comparison to the wt strain (*p* < 0.05). Data are means of at least three independent experiments (±SD).

We also studied the ability of gonococcal mutants concerning the Type I M.NgoAV to attach and invade human epithelial cells. Monolayers of human Hec-1-B cells were infected with gonococci for 4 h. The number of total bacterial cells (total CFU) added to the Hec-1-B cells, was enumerated. Then, bacteria that were attached to or have penetrated into the epithelial cells were released. Under our experimental conditions, the adhesion index of the wt strain was 2.39 (±0.68) as shown in Figure 6A. The differences in adhesion indexes between the studied mutant with knockout genes for M.NgoAV, NgoAVΔT mutant, and wt strain were not statistically significant. For NgoΔAV mutant, the adhesion index was 2.23 ± 0.68. The mutant NgoAVΔT has a decreased adhesion index to 1.12 ± 0.48 but *p* > 0.05.

Using the gentamycin resistance assay, we also calculated the invasion index, which allows determining the number of gonococcal cells that penetrate into the host human cells (Figure 6B). In our experimental conditions, the invasion index for wt gonococcal cells was 2.75 ± 1.94 × 10^4^. Inactivation of the M.NgoAV MTase (NgoΔAV mutant) did not influence the penetration abilities of mutant gonococci cells into human host cells (*p* > 0.05). For mutants with deleted M.NgoAV activity, the invasion index was 3.12 ± 1.47 × 10^4^. The shift of M.NgoAV specificity to M.NgoΔT decreased the invasiveness of *N. gonorrhoeae*: the invasion index was 1.4 ± 0.21 × 10^4^ (*p* < 0.05).

### Susceptibility of *Neisseria gonorrhoeae* to the presence of antibiotics and antimicrobials is different for wild-type and gonococcal variants in *ngoAV* locus

We adapted Kirby–Bauer disk diffusion susceptibility test to evaluate the toxicity of different antibiotics and chemical compounds for wt gonococci and their variants within *ngoAV* locus (Hudzicki, 2009). First, we measured the diameter of zones of inhibition of growth (in cm) to evaluate the effect of M.NgoAV disruption on antibiotic resistance of studied gonococci. The effect of polymyxin B or gentamycin on the growth of wt strain and variants within the locus encoding M.NgoAV was similar. In all cases, the diameters of zones of inhibition of growth were statistically not different between wt and mutant gonococci (*p* > 0.05) as shown in Table 2.

**TABLE 2:** Susceptibility of *N. gonorrhoeae* mutants within NgoAV RM system to antibiotics.

	Diameter of inhibition zone in cm					
	Cefotaxim 30μg/ml	Imipenem 10μg/ml	Polymyxin 300μg/ml	Azithromycin 15μg/ml	Gentamycin 30μg/ml	Bacitracin 10μg/ml
wt	5.58 ± 0.17	4.65 ± 0.13	1.88 ± 0.15	4.18 ± 0.04	2.42 ± 0.10	2.48 ± 0.15
NgoΔAV	**4.85 ± 0.19**	**4.35 ± 0.06**	1.9 ± 0.08	**4.50 ± 0.12**	2.48 ± 0.21	**2.80 ± 0.10**
NgoAVΔT	5.48 ± 0.13	**4.38 ± 0.04**	1.85 ± 0.10	4.28 ± 0.13	2.3 ± 0.14	**2.00 ± 0**
compAV	5.24 ± 0.17	4.54 ± 0.25	1.85 ± 0.06	4.18 ± 0.04	2.53 ± 0.05	2.6 ± 0.14

Significantly different values, comparing to wt strain (*p* < 0.05) are bolded.

The absence of active M.NgoAV (the knockout mutant NgoΔAV) caused a decrease in the susceptibility to imipenem and cefotaxime (4.35 ± 0.06 and 4.85 ± 0.19 cm, respectively) as compared to the values obtained for wt strain (4.65 ± 0.13 and 5.58 ± 0.17, for imipenem and cefotaxime, respectively; *p* < 0.05). For this mutant, we also observed the increase of susceptibility to azithromycin and bacitracin (4.50 ± 0.12 and 2.80 ± 0.10, respectively) as compared to values obtained for wt strain (4.18 ± 0.04 and 2.48 ± 0.15, for azithromycin and bacitracin, respectively; *p* < 0.05).

Application of imipenem had a similar effect on the mutant with deleted MTase (NgoΔAV) and on the mutant with changed specificity (NgoAVΔT): the size of inhibition zones decreased for imipenem (4.38 ± 0.04) compared to values observed for wt strain (4.65 ± 0.13; *p* < 0.05), suggesting that the effect may be due to the lack of M.NgoAV-specific methylation. M.NgoAVΔT mutant has the same sensitivity to azithromycin as wt strain (4.28 ± 0.13 and 4.18 ± 0.04, respectively; *p* < 0.05). The exposition to cefotaxime did not affect the value of the diameter of inhibition zones for NgoAVΔT mutant (5.48 ± 0.13) compared to wt strain (5.58 ± 0.17; *p* > 0.05). The susceptibility of NgoAVΔT to bacitracin was lower than those of wt strain (2.0 ± 0.01 and 2.48 ± 0.15, for NgoAVΔT and wt, respectively; *p* < 0.05), whereas the susceptibility of knockout mutant NgoΔAV to bacitracin was higher than those of wt strain (2.8 ± 0.10; *p* < 0.05).

The introduction of wt copy of *hsdS1* gene restores the values similar to those obtained for wt gonococci for all antibiotics tested.

Second, we tested the susceptibility of *N. gonorrhoeae* to different compounds with antimicrobial activity.

The treatment with SDS caused the formation of inhibition zones of about 1.5 cm on all tested gonococci: wt and mutants within M.NgoAV (Table 3). H_2_O_2_ inhibited wt *N. gonorrhoeae* growth and the diameter of the inhibition zone was 4.25 ± 0.27. The effect was similar for NgoΔAV mutant and complementant strain (4.20 ± 0.30 and 4.20 ± 0.18, respectively), but NgoAVΔT mutant was more susceptible to H_2_O_2_ action (4.78 ± 0.24; *p* < 0.05) compared to wt strain. The inhibition zones for wt gonococci and complementant strain were comparable when Triton X-100 was applied (2.46 ± 0.16 and 2.48 ± 0.13, respectively; *p* > 0.05). Deletion of M.NgoAV encoding genes (NgoΔAV mutant) or change of M.NgoAV specificity (NgoAVΔT) caused a slight resistance to Triton X-100 compared to wt strain: the values of the size of inhibition zone were 2.25 ± 0.23 and 2.13 ± 1.7 respectively (*p* < 0.05).

**TABLE 3:** Susceptibility of *N. gonorrhoeae* mutants within NgoAV RM system to chemicals.

	Diameter of inhibition zone in cm		
	30% H_2_O_2_	10% SDS	Triton X-100
wt	4.25 ± 0.27	1.53 ± 0.05	2.46 ± 0.16
NgoΔAV	4.20 ± 0.3	1.55 ± 0.06	**2.25 ± 0.23**
NgoAVΔT	**4.78 ± 0.24**	1.50 ± 0.04	**2.13 ± 0.17**
compAV	4.20 ± 0.18	1.50 ± 0.01	2.48 ± 0.13

Significantly different values, comparing to wt strain (*p* < 0.05) are bolded.

### Impact of the M.NgoAV inactivation on gonococcal transcriptome

**FIGURE 7:**
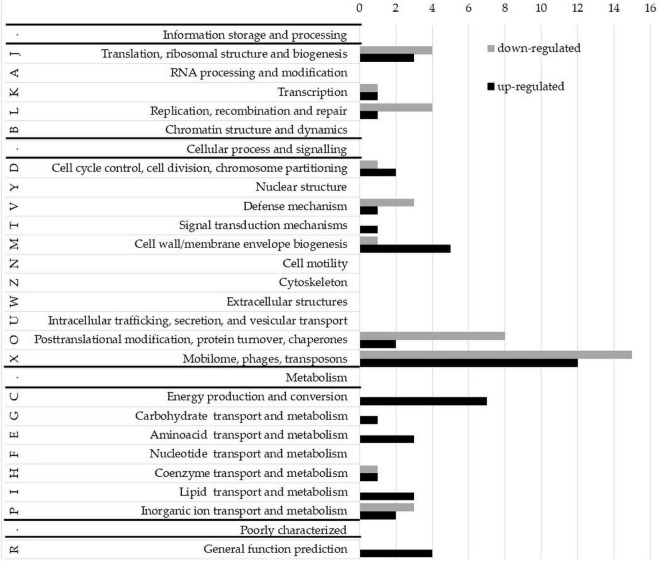
Clusters of orthologous groups (COG) classification of proteins encoded by upregulated and downregulated genes in the NgoAV knock-out *N. gonorrhoeae* (NgoΔAV) vs. the wt *N. gonorrhoeae* FA1090 strain. Letters represent COGs categories; numbers–the number of proteins in each category; black bars–number of proteins encoded by upregulated genes; gray bars–number of proteins encoded by downregulated genes. S category (function unknown) is not presented.

To evaluate whether phase-variable Type I NgoAV MTase regulates gene expression, the transcriptomes of wt *N. gonorrhoeae* FA1090 strain and NgoΔAV mutant within the locus encoding NgoAV were compared by RNA microarrays. A fold regulation cutoff of 2.0 was selected for the analysis. In the NgoAV knockout mutant (NgoΔAV), a total of 156 genes were deregulated (64 downregulated and 92 upregulated) as compared to the wt strain under the standard growth conditions by microarrays (Supplementary Table 2). This group of genes represented 7.23% of the total gene pool of *N. gonorrhoeae* FA1090 strain. For each gene with deregulated expression in NgoΔAV mutant vs. wt strain, we determined the COG (cluster of the orthologous gene) category (Galperin et al., 2021; Figure 7; Supplementary Table 2).

Most of the deregulated gonococcal genes (70 genes) encode proteins with unknown functions (S-function unknown COG category; Supplementary Table 2).

Yet, the most representative group of deregulated genes classified in COG concern genes from X category (mobilome: phages, transposons): 15 genes from this category had lower expression levels and 12 higher. The genome of *N. gonorrhoeae* FA1090 contains five genomic regions (NgoΦ1–5) that are related to dsDNA lysogenic phage and four regions (NgoΦ6–9) corresponding to filamentous phages (Piekarowicz et al., 2006, 2007). The downregulated genes are part of NgoΦ1-NgoΦ6 and NgoΦ8 phages, and the upregulated genes only of dsDNA phages NgoΦ1, NgoΦ2, NgoΦ4, and NgoΦ5. Most genes are coding uncharacterized phage-associated proteins, which have an unknown impact on the gonococcal phenotype.

A total of seventeen genes, encoding proteins involved in cellular metabolism, energy production, and conversion and transport (categories C, G, E, H, I, and P), were found to be upregulated in NgoΔAV mutant compared to wt cells (Figure 7, Supplementary Table 2). Encoded proteins are involved in the transport of amino acids, sugars, lipids, or other growth metabolites into the cell, suggesting higher metabolic activity of the variant. The function of some of these genes, as well as the possible significance of the deregulation of their expression, is hypothesized in the Discussion.

Concerning J category (translational, ribosomal structure, and biogenesis) four genes were downregulated and three are upregulated. In total, four genes related to RM systems were downregulated in NgoΔAV mutant compared to wt strain. Gene encoding Type II restriction endonuclease NgoAVII (NGO_0364) is upregulated 4.6 times.

Detailed information about deregulated genes can be found in Supplementary Table 2.

The expression of randomly chosen genes (determined by microarrays) was also investigated in NgoΔAV, NgoΔT, and compNgoAV variants by qRT-PCR (Supplementary Table 3). Most of the differences in expression between wt and knockout mutant measured by microarrays were confirmed by qRT-PCR. Moreover, the results suggested the differences between mentioned variants and NgoΔT mutant transcriptomes indicating the existence of different pools of genes whose expression is regulated by Type I MTase with its phase-variable specificity (Supplementary Table 3). For example, according to qRT-PCR, the expression of NGO_834, NGO_835, NGO_1804, and NGO_1547 genes was upregulated in NgoΔAV and downregulated in NgoΔT compared to wt strain with wt M.NgoAV MTase. On contrary, NGO_1046 gene expression was downregulated in NgoΔAV and upregulated in NgoΔT compared to wt strain.

## Discussion

In the present work, we have demonstrated for the first time that the activity and specificity of the phase-variable Type I RM system, NgoAV, affect the phenotype and global gene expression of *N. gonorrhoeae* FA1090.

The regulation of gene expression controlled by Type I MTases was suspected for a long time. Recently, the effect of inactivation of archetypal Type I RM encoded by *E. coli* on gene expression was investigated. Authors demonstrated that the inactivation of three Type I MTases in different *E. coli* strains had no impact on gene regulation or on cellular phenotype among >1,000 growth conditions tested (Mehershahi and Chen, 2021). The data suggest that the canonical Type I RM systems are involved in host defense mechanisms, without secondary regulatory functions in host physiology and virulence. However, the archetypal Type I RM systems are non-phase-variable as most known Type I systems. The screening of sequences encoding Type I RM systems deposited in REBASE [database of RM systems (Roberts et al., 2015)] revealed that DNA sequence repeats that mediate PV of gene expression by slipped−strand mispairing are found in 7.9% of *hsdS*, 2% of *hsdM*, and of 4.3% of *hsdR* genes (Atack et al., 2020). The PV of Type I MTase/endonuclease expression or specificity may lead to the control of the phasevarion expression as in the case of Type III MTases. However, less was known about the regulation of gene expression by phase-variable Type I MTases. The first phase-variable Type I RM system has been discovered in *Mycoplasma pulmonis* (Sitaraman and Dybvig, 1997). The PV allows for the generation of four different specificity types but the effect on gene expression has not been studied (Sitaraman and Dybvig, 1997). Authors proposed that variations in the production of RM activity are important for the survival of the *M. pulmonis* within the host (Gumulak-Smith et al., 2001). In the genome of *Listeria monocytogenes*, the presence of two distinct *hsdS* genes was confirmed, allowing for the generation of four possible specificities of Type I RM system but the impact on a potential phasevarion was not studied (Fagerlund et al., 2016). Data suggest that Type I RM PV may be involved in the regulation of virulence during the acute phase of meningitidis caused by this pathogen (Zbinden et al., 2020). A report describes the inactivation of the *hsdS* gene in *H. pylori*, which leads to the changes in the expression of some bacterial genes, suggesting the involvement of a Type I MTase in the control of gene expression. However, the global transcriptome of this mutant with inactivated Type I MTase was not studied (Furuta et al., 2014). The best-documented example of PV and gene expression regulation was observed in *Streptococcus pneumoniae*, where some strains carry a phase-variable Type I RM system SpnD39III. The specificity switch of the HsdS subunit of SpnD39III is driven by DNA inversions and can result in up to six distinct methylation specificities (Manso et al., 2014; Li et al., 2016). The variation in methylation patterns has an impact on global epigenetic changes. SpnD39III system appears to be a central regulatory mechanism improving the fitness of the *S. pneumoniae* in distinct host niches (Manso et al., 2014). The phase variation of SpnD39III is specifically associated with either invasive disease (lung infection) or nasopharyngeal carriage *in vivo* in a mouse model (Oliver et al., 2017). Other pathogenic and non-pathogenic bacteria, as *Lactobacillus salivarius*, also encode phase-variable Type I RM systems, but the impact of their PV on global transcriptome remains unknown (Claesson et al., 2006).

Our research model organism, *N. gonorrhoeae* FA1090, encodes 16 different MTases, two of which belong to Type I RM system [REBASE (Roberts et al., 2015)]: phase-variable NgoAV and non-phase-variable NgoAVIII. The PV of NgoAV system involves the switch of the specificity of MTase. M.NgoAV recognizes the interrupted, quasipalindromic sequence 5′-GCA(N_8_)TGC-3′ and methylates both DNA strands. M.NgoAVΔ recognizes 5′-GCAN_7_GTCA-3′ and 5′-GCAN_7_CTCA-3′, but the latter sequence is methylated only on one strand (Adamczyk-Poplawska et al., 2011). *In silico* analysis demonstrated that M.NgoAV may methylate 916 sites within the genome of *N. gonorrhoeae* FA1090 (AE004969) and M.NgoAVΔT only 340 sites. We, therefore, have hypothesized that complete removal of M.NgoAV activity would affect gene expression, fitness, and virulence of studied gonococci. Deletion of M.NgoAV actually induced global changes in the transcriptome of the studied variant. The upregulation and downregulation of gene expression levels had consequences on the altered phenotype of studied bacteria (NgoΔAV and NgoAVΔT vs. wt strain). For the first time, the existence of the phasevarion driven by the phase-variable Type I MTase has been strongly suggested. However, the determination of genes belonging to the phase-variable transcriptomes should be further investigated.

The alteration of gene expression, demonstrated in NgoΔAV mutant after 24 h of growth, had also a reflection on the phenotype of studied variants. Deletion of M.NgoAV resulted in an enhanced planktonic form of growth rather than the formation of biofilm. The shift of NgoAV specificity to NgoAVΔT has no effect on the biomass of biofilm as compared to wt strain. Surprisingly, the reinsertion of *hsdS1* gene did not restore the wt growth after 24 h: the biofilm formed by compNgoAV variant had then reduced OD_570/600_ ratio, comparable to the NgoΔAV mutant. In the compNgoAV variant, the *hsdS1* gene is under the control of a strong constitutive promoter P_opa_ as previously described (Ramsey et al., 2012), whereas the natural promoter of NgoAV is unknown. We could not exclude that in the case of complementation mutant, strong expression of *hsdS1* gene from P_opa_ causes variance of phenotype. We have then measured the level of mRNA encoding for the HsdS1 subunit in wt and complementant strain (Supplementary Figure 1; Supplementary Table 3). Our results suggest the synthesis of both, the functional wt subunit and the non-functional fragments of the HsdS1 subunit, in the compNgoAV variant. Such situation is due to the fact of interruption of *hsdS1* gene by insertion of *cm* cassette into the EcoRV site (Supplementary Figure 1). The existence of non-functional HsdS1 fragments may perturb the proper functioning of M.NgoAV in complementant strain by competition of non-functional HsdS::cm with wt HsdS for the HsdM subunit. Mernagh et al. (1997) had demonstrated that the HsdM subunit, in addition to its catalytic role in the MTase activity, is important for DNA binding. Physiologically, the expression of M.NgoAV-forming subunits may be tightly regulated, and differences in the concentration of HsdM and wt HsdS subunits in complementant cells may affect the MTase activity.

The observation of biofilm under FE SEM also showed that biofilms formed by wt strain and NgoΔAV mutant are comparable. NgoAVΔT mutant cells forming biofilm looked like they were experiencing problems maintaining their shape: cells seemed to collapse during the preparation of samples for microscopy. The biofilm formed by NgoAVΔT mutant cells appears flat and the cells size is very large. The disruption in biofilm formation and cell shape is also seen in the case of compNgoAV mutant. The measure of the area of cells forming biofilms indicated that in the case of NgoAVΔT and compNgoAV, cells were larger than that wt or NgoΔAV cells. However, there was a statistical difference between the size of cells forming NgoAVΔT and compNgoAV biofilms: compNgoAV cells forming biofilm were much smaller. Once again, the results suggest that the precise control of the expression level of genes encoding subunits forming M.NgoAV may have an impact on biofilm formation.

As we mentioned above, the expression of seven genes from C category (energy production and conversion) was upregulated, and this may have influence the biofilm formation ability by NgoΔAV variant. For example, the expression of *ldhA* gene (NGO_2043) coding lactate dehydrogenase is upregulated in NgoΔAV mutant. In closely related *N. meningitidis*, deletion of this gene promotes biofilm formation (Sigurlásdóttir et al., 2019). Moreover, the NGO_1751 gene (also C category) from *nuo* operon (encoding proteins involved in respiration with NADH as an electron donor) is upregulated in NgoΔAV mutant and was described as more highly expressed during planktonic growth of gonococci (Falsetta et al., 2009). Another gene from C category, upregulated over 5-folds, is *lctP* (NGO_1449) encoding lactate permease. It was described that *lctP* gonococcal mutant was significantly attenuated in its ability to colonize and survive in the murine genital tract (Exley et al., 2007). On the contrary, the expression of *mntC* (NGO_0168, P category), encoding a periplasmic divalent cation binding receptor protein, MntC, is downregulated in NgoΔAV. It has been demonstrated that *mntC* mutants of *N. gonorrhoeae* showed a reduced ability to form a biofilm. MntC was also involved in the protection of *N. gonorrhoeae* against oxidative stress (Lim et al., 2008).

Other genes whose products are engaged in response to stress were also downregulated in the NgoΔAV variant, according to microarrays: eight downregulated genes’ code for proteins engaged in O category (post-translational modifications, chaperones), including four chaperones *clpB* (NGO_1046), *groEL* (NGO_2095), *groES* (NGO_2094), and *hsp33* (also called hlsO, NGO_1189). Chaperone ClpB is involved in protein homeostasis. *GroES* and *groEL* genes are arranged in a bicistronic operon in *N. gonorrhoeae* and encode chaperones induced by stress conditions (Gunesekere et al., 2006). *Hsp33* gene encodes heat shock chaperonic protein. The comparison of protein expression in biofilm and planktonic gonococcal cells has demonstrated that the levels of ClpB and GroEL were reduced in cells forming biofilm (Phillips et al., 2012). In our study, after 24 h of growth, we observed the downregulation of these four genes and enhanced planktonic growth of NgoΔAV mutant vs. wt strain. Another downregulated gene from O category, *glr3* (NGO_0114), also encodes a protein involved in resistance to oxidative stress: glutaredoxin. The *ctpA/prc* gene (NGO_0572, still O category) encoding a C-terminal protease was also downregulated in NgoΔAV. Inactivation of this gene in *E. coli* resulted in reduced heat-shock response (thermal and osmotic stress) (Hara et al., 1991). So, in the NgoΔAV mutant, we can observe the deregulation of genes encoding proteins involved in response to stress. These results suggested that the NgoΔAV mutant may be more susceptible to stress than the wt strain. This hypothesis was also formulated after the studies of gonococcal variants within the NgoAV locus, in regard to their resistance to tested antimicrobials.

Application of 30% H_2_O_2_ and 10% SDS had the same inhibitory effect on NgoΔAV mutant and wt strain growth, but Triton X100 seemed to be more toxic to NgoΔAV mutant than to wt gonococci. NgoAVΔT variant also had increased susceptibility to H_2_O_2_ compared to wt cells. The resistance to SDS is related to *mtrCDE* expression (Chitsaz et al., 2019), which is not affected by the deletion of the M.NgoAV locus. Triton X-100 resistance of gonococci is regulated among others by the activity of transcriptional factor encoded by *araC* (NGO_0025) (Rouquette et al., 1999), which was dysregulated in NgoΔAV knockout mutant. Therefore, our phenotypic observations are concordant with the microarray transcriptomic data.

The observation of susceptibility of mutant gonococci in M.NgoAV locus (NgoΔAV) to antibiotics had demonstrated that the knockout mutant was more susceptible to bacitracin but less susceptible to cefotaxime and imipenem than the wt strain. Bacitracin blocks the transport of peptidoglycan subunits across the cytoplasmic membrane. Cefotaxime and imipenem interact directly with penicillin-binding proteins (PBPs) and inhibit transpeptidase activity. We have also observed the upregulation of the *rlpA* (NGO_1728) gene and the downregulation of the *envc* (NGO_0571) gene in the NgoΔAV mutant compared to the wt strain. The *rlpA* gene encodes peptidoglycan-degrading enzymes (peptidoglycan lytic transglycosylase). It was demonstrated for *E. coli* that the deletion of the *rplA* gene causes supersensitivity to bacitracin (Kamata and Matsuhashi, 2017). The *envc* product is an activator of murein hydrolases. Deregulation of the expression of both these genes affects the peptidoglycan turnover, and this may alter the susceptibility of bacteria to antibiotics acting on peptidoglycan.

In total, 27 regulated genes (15 downregulated and 12 upregulated) belong to the X category (mobilome, phages, and transposons). Most of them are prophage genes. It was previously demonstrated that distinct *N. gonorrhoeae* phage-associated gene expression patterns are detected during genital infection in men and women. Phage-associated genes were significantly upregulated in gonococci isolated from female cervicovaginal lavage samples (Nudel et al., 2018). While female specimens had higher expression of a broad range of phage types (both double-stranded DNA and filamentous, single-stranded DNA phages), male specimens (male urethral samples) had higher expression only in the subset of dsDNA prophages (Nudel et al., 2018). The role of phage genes in *N. gonorrhoeae* infection has not been studied extensively, but their expression was demonstrated to be increased in female subjects and suspected to relate to the formation of biofilms by gonococci in the female genital tract. In NgoΔAV mutant, only genes of dsDNA phages were enriched, like in gonococci isolated from male samples and mutant cells form a biofilm with lower biomass than wt strain.

Our *in silico* analysis pointed out the existence of three main groups of gonococci: (i) with functional HsdS1 subunit (like M.NgoAV in FA1090 strain), (ii) with the fused form (like in NgoΔT variant), or (iii) with no active NgoAV-like MTase (like NgoΔAV variant). We made an effort to find an association between the form of MTase and the isolation source of gonococci. It seems that in men, more often, the fused form or no M.NgoAV-like may be found, while in the female, the HsdS1 form is more present. The role of the PV of M.NgoAV activity and/or specificity in the invasiveness of different niches (female genital tract or male urogenital tract, but also other tissues) by gonococci should be further investigated as well as the involvement of prophages genes in this process.

Concerning the interaction of gonococci with host human cells, deletion of *hsdS1* has no effect on adhesion or invasion of human endocervical cell line by mutant gonococci, despite the upregulation of genes encoding OpaD and porin P.IB, both suspected to promote invasion of host cells. The shift of specificity was accompanied by a decrease in the number of cells invading the human host cell cytoplasm. These observations suggest that methylation of sites recognized by NgoAVΔT has an impact on the expression of unknown factors that regulate the penetration of gonococci through the cellular membrane.

The rapidly growing antibiotic resistance of gonococci is currently a major public health concern. The unusual adaptation ability of gonococci to different environmental niches reflects a huge phenotypic variation in gonococcal cells (Cohen and Sparling, 1992). The knowledge of mechanisms ruling such adaptability to different growth conditions is indispensable for developing new anti-gonococcal drugs. Our results suggest that phase-variable Type I MTase may play an important role in the variability of *N. gonorrhoeae* and therefore may be the target of a new cure against this pathogen.

In conclusion, we have demonstrated for the first time that alteration in the activity or the specificity of phase-variable Type I MTase M.NgoAV has an impact on the phenotype of gonococci. Perturbation of M.NgoAV activity had mainly an impact on biofilm formation, favoring planktonic growth. Second, the PV of M.NgoAV specificity resulted in the alteration of the susceptibility of gonococci to studied antibiotics acting on the peptidoglycan biosynthesis. The PV of M.NgoAV also disturbed the invasion of host human epithelial cells. For the first time, the existence of a group of genes whose expression is directed by a phase-variable NgoAV was pointed out, suggesting the existence of a phasevarion controlled by phase-variable Type I MTase. Our observations also suggest a differential distribution of M.NgoAV forms (HsdS1 or fused or inactive) between men and women. Deletion of M.NgoAV activity resulted in disparate regulation of phage-associated genes, which may play a role in the colonization of men’s and women’s urogenital tracts. M.NgoAV would be implicated in the control of gonococcal virulence and spread during infection and perhaps in better colonization of a niche. Further studies are necessary, for a better understanding of the role of phase-variable M.NgoAV in *N. gonorrhoeae* physiology; for example, the comparison of M.NgoAV and NgoAVΔT phasevarions would be undertaken.

## Data availability statement

The datasets presented in this study can be found in online repositories. The names of the repository/repositories and accession number(s) can be found in the article/Supplementary material.

## Author contributions

MA-P and AP: conceptualization. PB and AK: SEM. MA-P: writing – original draft preparation, project administration, and funding acquisition. MA-P, AP, and AK: writing – review and editing. MA-P, AM, and NM: investigation. MA-P and AK: data analysis. All authors contributed to the article and approved the submitted version.
