# Supplementary material for: Phase-variable Type I methyltransferase M.NgoAV from Neisseria gonorrhoeae FA1090 regulates phasevarion expression and gonococcal phenotype
Source: Front Microbiol. 2022 Oct 4;13:917639. doi: 10.3389/fmicb.2022.917639 (PMC9577141; doi:10.3389/fmicb.2022.917639)
Supplement: Supplementary file 1 [file Data_Sheet_1.PDF]

Supplementary Material

1 Supplementary Figures and Tables

1.1 Supplementary Tables

1.1.1 Table S1. List of *N. gonorrhoeae* strains, encoding *ngoAV* locus, according to the number of guanine in polyG tract and the probability of encoded functional MTase.

	Strain	Functional form	Isolation source
**6 guanines**			
1	34769	fusion	endocervix
2	NG196	fusion	urethral discharge
3	NG290	fusion	vaginal swab
4	NG251	fusion	urethral swab
5	FQ36	fusion	urethra
6	32867	fusion	unknown
7	NJ189125	functional HSDS2	urine
9	FQ82	fusion	rectum
10	FQ04	fusion	rectum
11	FQ35	fusion	urethra
12	FQ84	fusion	pharynx
13	TFG-A2	fusion	throat
14	TFG-B2	fusion	throat
15	FQ20	fusion	urethra
**7 guanines**			
1	1291	HsdS1	unknown

2	3648	HsdS1	unknown
3	4020	none	unknown
4	Feb-35	none	unknown
5	34530	none	urethra
6	88G285	HsdS1	disseminated infection
7	98D159	none	disseminated infection
8	BJ16148	none	unknown
9	CT213	HsdS1	urethra
10	FA1090	HsdS1	endocervix/disseminated
11	FC428	none	unknown
12	FC460	none	unknown
13	FC498	none	unknown
14	FDAARGOS_204	none	cervix
15	FDAARGOS_205	none	unknown
16	FDAARGOS_207	HsdS1	disseminated infection
17	FQ48	none	urethra
18	MS11	HsdS1 two copies	urethritis
19	NCCP11945	none	unknown
20	NCTC13484	none	urogenital
21	NCTC13798	none	throwat swab
22	NCTC13799	none	throat swab
23	NCTC13800	none	throat swab
24	NG-k51.05	none	unknown/male

25	NJ1711654	none	urethral discharge
26	O1G1370	none	disseminated infection
27	O2D156	none	disseminated infection
28	RIVM0610	none	unknown
29	RIVM0640	none	unknown
30	SRRSH203	none	urine
31	SRRSH204	none	urine
32	SRRSH205	none	urine
33	SRRSH207	none	urine
34	SRRSH214	none	urine
35	SRRSH229	none	urine
36	SRRSH240	none	urine
37	SS3160	none	urine
38	SW0002	HsdS1	vaginal discharge
39	SW0005	HsdS1	vaginal discharge
40	SW0011	HsdS1	vaginal discharge
41	SW0015	HsdS1	vaginal discharge
42	SW0018	HsdS1	vaginal discharge
43	SW0032	HsdS1	vaginal discharge
44	SW0223	HsdS1	vaginal dicharge
45	SW0236	HsdS1	vaginal discharge
46	SW0259	HsdS1	vaginal discharge
47	SW0274	none	vaginal discharge

48	TUM 19855	none	unknown
49	TUM16691	none	unknown
50	TUM19854	none	unknown
51	WHO F	HsdS1	unknown
52	WHO K	none	unknown
53	WHO L	none	unknown
54	WHO O	HsdS1	unknown
55	WHO P	none	uretrogenital
56	WHO_V	none	urethral
57	WHO_W	HsdS1	unknown
58	WHO_X	none	pharynx
59	WHO_Y	none	urethra
60	WHO_Z	none	female genital swab
**8 guanines**			
1	TUM15748	none	unknown
2	FA19	HsdS1	disseminated infection
3	AUSMDU00010541	HsdS1	unknown
4	FQ02	HsdS1	rectum
5	SK92679	HsdS1	disseminated infection
6	G7944	HsdS1	unknown
7	G97687	HsdS1	unknown
**9 guanines**			
1	TUM15753	fusion	unknown

2	TUM19853	fusion	unknown
3	WHO U	fusion	pharengeal specimen
4	WHO N	fusion	unknown
5	WHO G	fusion	unknown
6	20181204	fusion	unknown
7	FQ01	fusion	pharynx
8	WHO M	fusion	unknown
9	F18	fusion	vagina
10	FA640	fusion	unknown

1.1.2 Table S2. Differentially expressed genes in the NgoΔAV knock-out *N. gonorrhoeae* mutant (*ngoaVhsdS1::cm*) *versus* wild-type *N. gonorrhoeae* FA1090a according to microarray test. Identity of the genes is indicated by gene and protein accession numbers, according to NCBI in the annotation of the *N. gonorrhoeae* FA1090 genome. The presented average ratio is the mean of *N. gonorrhoeae* mutant/ wild type *N. gonorrhoeae* FA1090. Only those genes with an expression ratio above 2.0-fold and *P* < 0.05 were included in this study. Protein functions were assigned according to BLAST and Refseq databases. COGs were assigned with the NCBI Database of Clusters of Orthologous Genes (COGs) https://www.ncbi.nlm.nih.gov/research/cog/.

ORF	Gene name or phage	Protein	function	Fold change	COG number	COG category
**down**						
NGO_0025	araC	YP_207206.1	AraC-type DNA-binding domain and AraC-containing proteins, transcriptional regulator	-2.75	2207	K
NGO_0049		YP_207228.1	hypothetical protein	-2.16		
NGO_0114	glr3	YP_207286.1	Glutaredoxin	-2.24	0695	O
NGO_0122		YP_207294.1	hypothetical protein	-2.13		

NGO_0167		YP_207335.1	hypothetical protein	-2.76		
NGO_0168	mntC	YP_207336.1	ABC-type Zn uptake system ZnuABC, Zn-binding component ZnuA	-2.26	0803	P
NGO_0227		YP_207392.1	hypothetical protein	-2.02		
NGO_0311		YP_207470.1	hypothetical protein	-2.03		
NGO_0389		YP_207545.1	Pseudouridylate synthase RsuA, specific for 16S rRNA U516 and 23S rRNA U2605	-2.05	1187	J
NGO_0465		YP_207615.1	hypothetical protein	-2.07		
NGO_0498	NgoΦ1	YP_207648.1	putative phage associated protein	-2.47		X
NGO_0499	NgoΦ1	YP_207649.1	putative phage associated protein	-2.23		X
NGO_0500	NgoΦ1	YP_207650.1	putative phage associated protein	-2.02		X
NGO_0503	NgoΦ1	YP_207653.1	putative phage associated protein	-2.19		X
NGO_0555		YP_207701.1	hypothetical protein	-4.28		
NGO_0571	envC	YP_207716.1	Septal ring factor EnvC, activator of murein hydrolases AmiA and AmiB	-2.09	4942	D
NGO_0572	ctpA	YP_207717.1	C-terminal processing protease CtpA/Prc, contains a PDZ domain	-2.20	0793	O
NGO_0580		YP_207723.1	Thioredoxin reductase	-2.04	0492	O
NGO_0633	nifU	YP_207773.1	Fe-S cluster assembly scaffold protein IscU, NifU family	-2.01	0822	O
NGO_0638		YP_207777.1	hypothetical protein	-2.87		
NGO_0640	rmsR	YP_207779.1	Type III restriction endonuclease	-2.09	3421	V
NGO_0641		YP_207780.1	Adenine specific DNA methylase Mod	-2.54	2189	L
NGO_0654		YP_207793.1	NH3-dependent NAD+ synthetase	-2.12	0171	H
NGO_0660	gatB	YP_207799.1	Asp-tRNAAsn/Glu-tRNAGln amidotransferase B subunit	-2.26	0064	J
NGO_0698		YP_207834.1	hypothetical protein	-2.88		

NGO_0699	hsdS	YP_207835.1	Restriction endonuclease S subunit	-3.42	0732	V
NGO_0701		YP_207837.1	hypothetical protein	-3.51		
NGO_0725	NgoΦ5	YP_207857.1	putative phage associated protein	-4.26		X
NGO_0873		YP_207986.1	DNA-cytosine methylase	-2.17	0270	L
NGO_0908	fitA	YP_208018.1	Plasmid stability protein StbC1, contains ribbon-helix-helix domain	-2.04	4691	V
NGO_0982		YP_208080.1	hypothetical protein	-2.24		
NGO_0998	dnaG	YP_208093.1	DNA primase (bacterial type)	-2.56	0358	L
NGO_1014	NgoΦ4	YP_208109.1	putative phage associated protein	-2.80		X
NGO_1015	NgoΦ4	YP_208110.1	putative phage associated protein	-2.24		X
NGO_1046	clpB	YP_208130.1	ATP-dependent Clp protease, ATP-binding subunit ClpA, ClpB	-2.48	0542	O
NGO_1069		YP_208151.1	hypothetical protein	-2.41		
NGO_1072		YP_208153.1	hypothetical protein	-2.57		
NGO_1084		YP_208163.1	hypothetical protein	-2.51		
NGO_1095	NgoΦ2	YP_208174.1	putative phage associated protein	-2.09		X
NGO_1104	NgoΦ2	YP_208183.1	putative phage associated protein	-2.44		X
NGO_1145	NgoΦ6	YP_208224.1	putative phage associated protein	-2.58		X
NGO_1147		YP_208226.1	hypothetical protein	-2.29		
NGO_1170	NgoΦ8	YP_208248.1	putative phage associated protein	-2.14		X
NGO_1186		YP_208258.1	hypothetical protein	-2.10		
NGO_1189	hsp33	YP_208261.1	Redox-regulated molecular chaperone, HSP33 family	-2.35	1281	O
NGO_1199	hrpA	YP_208271.1	HrpA-like RNA helicase	-2.74	1643	J
NGO_1210		YP_208281.1	hypothetical protein	-2.37		
NGO_1613	NgoΦ3	YP_208653.1	putative integrase/recombinase, putative	-2.52		X

			phage associated protein			
NGO_1633	NgoΦ3	YP_208673.1	putative phage associated protein	-2.09		X
NGO_1637	NgoΦ3	YP_208677.1	putative phage associated protein	-2.21		X
NGO_1648	irg7	YP_208687.1	Transposase	-2.50	3547	X
NGO_1653		YP_208692.1	hypothetical protein	-2.37		
NGO_1708	dinG	YP_208741.1	Rad3-related DNA helicase DinG	-2.28	1199	L
NGO_1771		YP_208802.1	Small-conductance mechanosensitive channel	-2.40	0668	M
NGO_1804	fabZ	YP_208834.1	3-hydroxymyristoyl/3-hydroxydecanoyl-(acyl carrier protein) dehydratase	-2.12	0764	I
NGO_1847		YP_208880.1	hypothetical protein	-2.00		
NGO_1966		YP_208988.1	hypothetical protein	-2.78		
NGO_1967		YP_208989.1	hypothetical protein	-3.03		
NGO_1981	lecA	YP_209001.1	hypothetical protein, adhesin	-2.30		
NGO_1983	map	YP_209003.1	Methionine aminopeptidase	-2.08	0024	J
NGO_2092		YP_209105.1	ABC-type enterochelin transport system, periplasmic component	-2.08	4607	P
NGO_2093	fetA	YP_209106.1	Outer membrane receptor protein, Fe transport, enterobactin	-2.24	1629	P
NGO_2094	groES	YP_209107.1	Co-chaperonin GroES (HSP10)	-3.56	0234	O
NGO_2095	groEL	YP_209108.1	Chaperonin GroEL (HSP60 family)	-2.35	0459	O
**up**						
NGO_0015		YP_207196.1	hypothetical protein	2.68		
NGO_0024		YP_207205.1	hypothetical protein	2.73		
NGO_0105		YP_207278.1	Palmitoleoyl-ACP: Kdo2-lipid-IV acyltransferase (lipid A biosynthesis)	2.11	1560	I
NGO_0110		YP_207283.1	Mg2+/citrate symporter	3.58	2851	C

NGO_0135		YP_207305.1	Periplasmic beta-glucosidase and related glycosidases	2.07	1472	G
NGO_0137		YP_207307.1	hypothetical protein	3.82		
NGO_0143	nhaC	YP_207313.1	Na+/H+ antiporter NhaC/MleN	2.00	1757	C
NGO_0145		YP_207315.1	hypothetical protein	2.35		
NGO_0147		YP_207317.1	hypothetical protein	2.13		
NGO_0198	amtB	YP_207363.1	putative transporter, ammonium	2.42		
NGO_0364		YP_207520.1	restriction endonuclease R.NgoVII	4.59		V
NGO_0430		YP_207585.1	hypothetical protein	2.05		
NGO_0433		YP_207587.1	Pseudouridine synthase RluA, 23S rRNA- or tRNA-specific	2.26	0564	J
NGO_0448		YP_207600.1	hypothetical protein	2.18		
NGO_0480	NgoΦ1	YP_207630.1	putative phage associated protein	2.45		X
NGO_0490	NgoΦ1	YP_207640.1	putative phage associated protein	2.42		X
NGO_0495	NgoΦ1	YP_207645.1	Phage terminase large subunit	2.18	5362	X
NGO_0510	NgoΦ1	YP_207660.1	putative phage associated protein	2.26		X
NGO_0522	NgoΦ1	YP_207672.1	Phage-related minor tail protein	3.70	5281	X
NGO_0529		YP_207679.1	Choline-glycine betaine transporter	3.58	1292	M
NGO_0590	ftsK	YP_207733.1	DNA segregation ATPase FtsK/SpoIIIE or related protein,ftsK-like cell division/stress response protein	2.04	1674	D
NGO_0602	nmlR	YP_207742.1	DNA-binding transcriptional regulator, MerR family	2.40	0789	K
NGO_0606		YP_207746.1	Na+-dependent transporter, SNF family	3.61	0733	R
NGO_0631		YP_207771.1	hypothetical protein	3.02		
NGO_0672		YP_207811.1	hypothetical protein	2.08		
NGO_0711		YP_207843.1	Threonine dehydrogenase or related Zn-dependent dehydrogenase	2.02	1063	E

NGO_0712		YP_207844.1	hypothetical protein	3.21		
NGO_0723	NgoΦ5	YP_207855.1	putative phage associated protein	3.66		X
NGO_0724	NgoΦ5	YP_207856.1	putative phage associated protein	4.27		X
NGO_0796		YP_207922.1	hypothetical protein	3.34		
NGO_0802		YP_207928.1	hypothetical protein	2.70		
NGO_0806		YP_207932.1	Siroheme synthase (precorrin-2 oxidase/ferrochelatase domain)	2.21	1648	H
NGO_0834		YP_207955.1	Curli biogenesis system outer membrane secretion channel CsgG	2.20	1462	M
NGO_0835	GNA1162	YP_207956.1	Uncharacterized conserved protein, DUF799 domain	2.73	4380	S
NGO_0879		YP_207991.1	hypothetical protein	2.06		
NGO_0883		YP_207994.1	hypothetical protein	2.32		
NGO_0939		YP_208046.1	hypothetical protein	2.02		
NGO_0950		YP_208053.1	hypothetical protein	2.03		
NGO_0953		YP_208055.1	hypothetical protein	2.83		
NGO_0979		YP_208077.1	hypothetical protein	2.38		
NGO_1004	NgoΦ4	YP_208099.1	putative phage associated protein	2.16		X
NGO_1005	NgoΦ4	YP_208100.1	putative phage associated protein	2.02		X
NGO_1027		YP_208115.1	hypothetical protein	2.74		
NGO_1086	NgoΦ2	YP_208165.1	putative phage associated protein	3.23		X
NGO_1115	NgoΦ2	YP_208194.1	putative phage associated protein	2.03		X
NGO_1132	NgoΦ2	YP_208211.1	putative phage associated protein	2.95		X
NGO_1159		YP_208237.1	hypothetical protein	2.62		
NGO_1247		YP_208318.1	Phosphatidylglycerophosphate synthase	2.07	0558	I

NGO_1248		YP_208319.1	hypothetical protein	2.32		
NGO_1251		YP_208322.1	hypothetical protein	2.12		
NGO_1286	infB	YP_208352.1	Translation initiation factor IF-2, a GTPase	2.38	0532	J
NGO_1288		YP_208354.1	hypothetical protein	2.51		
NGO_1290		YP_208356.1	Na+/alanine symporter	2.19	1115	E
NGO_1297		YP_208363.1	Uncharacterized conserved protein, UPF0210 family	2.03	2848	D
NGO_1301		YP_208367.1	hypothetical protein	2.97		
NGO_1333		YP_208395.1	DNA gyrase/topoisomerase IV, subunit B	2.05	0187	L
NGO_1352		YP_208413.1	putative 3-oxoacyl-(acyl-carrier-protein) synthase	2.05		
NGO_1355		YP_208416.1	Na+-dependent transporter, SNF family	2.18	0733	R
NGO_1359		YP_208419.1	hypothetical protein	2.13		
NGO_1398		YP_208458.1	ABC-type transport system involved in multi-copper enzyme maturation, permease component	2.15	1277	O
NGO_1449	lctP	YP_208503.1	L-lactate permease	5.63	1620	C
NGO_1455		YP_208508.1	Mn2+ or Fe2+ transporter, NRAMP family	3.00	1914	P
NGO_1461		YP_208514.1	hypothetical protein	2.97		
NGO_1463		YP_208516.1	hypothetical protein	2.12		
NGO_1469		YP_208521.1	hypothetical protein	2.04		
NGO_1475		YP_208527.1	Predicted regulator of Ras-like GTPase activity, Roadblock/LC7/MglB family	2.06	2018	T
NGO_1497		YP_208547.1	hypothetical protein	2.78		
NGO_1513	opaD	YP_208563.1	Opacity protein LomR and related surface antigens OpaD	2.77	3637	M
NGO_1547		YP_208595.1	Undecaprenyl pyrophosphate	2.10	1968	I

			phosphatase			
NGO_1551		YP_208599.1	hypothetical protein	2.19		
NGO_1576		YP_208617.1	hypothetical protein	2.65		
NGO_1681		YP_208718.1	hypothetical protein	2.10		
NGO_1699		YP_208732.1	ABC-type uncharacterized transport system, permease component	2.19	4137	R
NGO_1728	rlpA	YP_208761.1	Peptidoglycan lytic transglycosylase RlpA, contains C-terminal SPOR domain	2.02	0797	M
NGO_1729		YP_208762.1	PepSY domain containing protein, regulator of zincin peptidase activity	2.04	3212	O
NGO_1751		YP_208784.1	NADH:ubiquinone oxidoreductase subunit 3 (chain A)	2.04	0838	C
NGO_1759		YP_208792.1	hypothetical protein	2.21		
NGO_1775		YP_208806.1	Na+-translocating ferredoxin:NAD+ oxidoreductase RNF, RnfB subunit	2.07	2878	C
NGO_1812	P.IB	YP_208842.1	Outer membrane porin OmpC/OmpF/PhoE, major outer membrane protein porin P.IB	2.21	3203	M
NGO_1861		YP_208894.1	hypothetical protein	2.35		
NGO_18781		YP_208911.1	hypothetical protein	2.44		
NGO_1887		YP_208916.1	hypothetical protein	2.07		
NGO_1888		YP_208917.1	hypothetical protein	2.51		
NGO_2008		YP_209028.1	Glycine/D-amino acid oxidase (deaminating)	2.12	0665	E
NGO_2043	ldhA	YP_209063.1	Lactate dehydrogenase or related 2-hydroxyacid dehydrogenase	3.58	1052	C
NGO_2090		YP_209103.1	ABC-type enterochelin transport system, permease component	2.88	4605	P
NGO_2096		YP_209109.1	Na+-dependent transporter, SNF family	2.08	0733	R
NGO_2113		YP_209125.1	Polyphosphate kinase 2, PPK2 family	2.13	2326	C

NGO_2136		YP_209145.1	hypothetical protein	3.99		
NGO_2142		YP_209151.1	hypothetical protein	2.18		
NGO_2179		YP_209184.1	hypothetical protein	2.10		
NGO_2181	rnpA	YP_209186.1	RNase P protein component	2.07	0594	J

1.1.3 Table S3. Differentially expressed genes in the NgoΔAV knock-out *N. gonorrhoeae* mutant (*ngoaVhsdS1::cm*), NgoΔT mutant with new specificity (*ngoAVhsdSΔT*) and compNgoAV variant (*ngoAVigatrpb::hsdS1*) *versus* wild-type *N. gonorrhoeae* FA1090. Identity of the genes is indicated by gene and protein accession numbers, according to NCBI in the annotation of the *N. gonorrhoeae* FA1090 genome. Genes were chosen randomly. Protein functions were assigned according to BLAST and Refseq databases. The presented average ratio is the fold change in expression of genes in *N. gonorrhoeae* FA1090 variant/wild type *N. gonorrhoeae* FA1090 as measured by qRT-PCR (*P* < 0.05). NS-not statistically significant.

ORF	Gene name	Protein	Function	Fold change comparing to wt strain		
				NgoΔAV	NgoΔT	Comp NgoAV
NGO_0105		YP_207278.1	Palmitoleoyl-ACP: Kdo2-lipid-IV acyltransferase (lipid A biosynthesis)	2.6	NS	NS
NGO_0590	ftsK	YP_207733.1	DNA segregation ATPase FtsK/SpoIIIE or related protein,ftsK-like cell division/stress response protein	2.04	NS	NS
NGO_0641		YP_207780.1	Adenine specific DNA methylase Mod	NS	NS	NS
NGO_0834		YP_207955.1	Curli biogenesis system outer membrane secretion channel CsgG	2.46	-1.8	NS
NGO_0835	GNA1162	YP_207956.1	Uncharacterized conserved protein, DUF799 domain	4,39	-1.41	NS
NGO_0873		YP_207986.1	DNA-cytosine methylase	-4.3	-5.0	NS
NGO_0908	fitA	YP_208018.1	Plasmid stability protein StbC1,	-1,61	-1.85	NS
NGO_1046	clpB	YP_208130.1	ATP-dependent Clp protease, ATP-binding subunit ClpA, ClpB	-1.5	3.85	NS

NGO_1189	hsp33	YP_208261.1	Redox-regulated molecular chaperone, HSP33 family	NS	1.2	NS
NGO_1449	lctP	YP_208503.1	L-lactate permease	7.19	NS	NS
NGO_1513	opaD	YP_208563.1	Opacity protein LomR and related surface antigens OpaD	1.35	NS	NS
NGO_1547		YP_208595.1	Undecaprenyl pyrophosphate phosphatase	2.46	-1.83	NS
NGO_1708	dinG	YP_208741.1	Rad3-related DNA helicase DinG	-2.11	-1.93	NS
NGO_1728	rlpA	YP_208761.1	Peptidoglycan lytic transglycosylase RlpA	4.43	NS	NS
NGO_1804	fabZ	YP_208834.1	3-hydroxymyristoyl/3-hydroxydecanoyl-(acyl carrier protein) dehydratase	2.36	-1.75	NS
NGO_1812	P.IB	YP_208842.1	Outer membrane porin OmpC/OmpF/PhoE, major outer membrane protein porin P.IB	2.99	1.27	NS
NGO_2094	groES	YP_209107.1	Co-chaperonin GroES (HSP10)	NS	3	NS
NGO_2095	groEL	YP_209108.1	Chaperonin GroEL (HSP60 family)	NS	3.85	NS
NGO_404	hsdM	YP_207560.1	Modification subunit from NgoAV	NS	NS	NS
NGO_407	hsdR	YP_207562.1	Restriction subunit from NgoAV	NS	NS	NS
NGO_406_3	hsdS1	WP_025456033.1	Specificity subunit from NgoAV	NS	NS	2.46
NGO_406_19	hsdS1	WP_025456033.1	Specificity subunit from NgoAV	NS	NS	2.26
NGO_406B	hsdS2	WP_012503463.1	Specificity subunit from NgoAV	NS	NS	NS

1.1.4 Table S4. List of primers used for gene amplification or qRT-PCR. For qRT-PCR primers, primer names are the same as the gene accession number.

**Primers for construction of mutant strains within locus encoding M.NgoAV**			
**Name**	**Forward primer**	**Reverse primer**	**Product size bp**

hsdSXylR /hsdSXylL	TAGAAGCTTGCTCGCCGCCTTG TCCTGAT	TACTGCAGACCACATGAACGCCA GCGAAC	see in text
NhehsdS/ SmahsdS	CTAGCTAGCAACAACCATGGAT ATGCAAAGCAAAGCG	TCCCCCGGGTTTTCAGACGGCAT TGATAGCCATCAGC	see in text
Smatrpb/ Nheiga	TCCCCCGGGGACTAACAAAGCT ACAGCTCAATGC	CTAGCTAGCGTCCGAAGCATGCA TGGAATGAC	6578
MAP16a/ MAP17a	ATCTGCAGGTGCTTGGGCGCCT TAGGGAATCGTTCTC	ATCTGCAGTTTGTACTGTCTGCG GCTCGCCGCCTTGTC	7041
MAP20/MAP21	GACTGCAGGGGTCAACCGCTAC GTCAAATG	CGAAAGCTTATCGGCGATTAACT CCGCTTCCAAC	4300
HsdSfor/HsdSrev	CGCGATCGGTTCCTGATAAGGG TATCCATAGAGAGCCTTCTATT ATCGTTAAATCAAGAG	AGACCATGGCAACCTCCCCCAAC GTCTTCCAAACCACATTTTTCAG ACGGCATTGATAGC	500
**Primers for qRT-PCR**			
**Primers for internal standard**			
**Name**	**Forward primer**	**Reverse primer**	**Product size bp**
16SRNA	GCGTGGGTAGCAAACAGGAT	CGCGTTAGCTACGCTACCAAG	81
**Primers for the study of mutant gonococci gene expression**			
**Name**	**Forward primer**	**Reverse primer**	**Product size bp**
Ngo0007	AATGGCAATCCAACCTGTTT	CGACAATGTGAATTCTTCGG	107
Ngo0105	AAGGCTTCGTGTTGCACAT	TATTCGGTATTGCGGTTGAA	91
Ngo0590	CGATGATCGTGGTCGTTATC	TGGCGACAATCATGTGTATG	126
Ngo0641	CCAATACATCGGCATTGAAC	CATAAACAAACTCGCCACCA	134
Ngo0834	GCAATACGCAATATCACGGT	GCAGGTGTGTTACCAGGATG	141
Ngo0835	GGTTACCGAATACGGCACTT	AACCCGACCACAACTCTTTC	107
Ngo0873	GGCTTGTCAAACCCTACGTT	CCGGCCAATAAATCAATACC	105
Ngo0908	AGTACCGAAGCAGAAATCCG	ATTTCCTGCCCTATTGATGC	98
Ngo1046	CGAATTTGAAGAACGCTTGA	GCCGACCAAAGTATGGATTT	97
Ngo1189	AGGTCAGCGATATGCTGTTG	ACGTATTTGCTGTGGCAGAA	106
Ngo1449	GCGTCTTACCAAACGCTGTA	TCCAGGCATCTTTGAATTTG	100
Ngo1513	CGGCAACCAGCTTAACATAA	GGGTTTGAATTTGTCGTTGA	124
Ngo1547	AACCGTTTCGTCCTCAATCT	ATGACTGCAACACTCAAGGG	113
Ngo1708	GCAAACAAATGCAGGATGTC	GCTTTGCCTTCTTCTATGGC	134
Ngo1728	TACAAAGTCGCCGAATTCAC	GCGGTAAAGGCGTTCATATC	110
Ngo1804	GTTTCAAACGCCAAGTCATC	CACATAATCACGGCTTCGAC	136

Ngo1812	CGACAATAATGCCCTGTACG	TGCTCCATACAATTTGGCAT	64
Ngo2094	TGACCATCCGTCCTTTACAC	CGATGACTTCGCCCATATC	123
Ngo2095	AAAGAAGGCGTGATTACCGT	CCGCGTCGTTGATAAAGTAA	115
**Primers for the study of expression of genes encoded by *ngoAV* locus**			
**Name**	**Forward primer**	**Reverse primer**	**Product size bp**
Ngo404	GATGACCGAAATGCAACAAC	AGTGTGCCGAGAACGTATTG	94
Ngo405	CACATCGACGAAACAGGCAA	CGCAAGCGGTTATGGCTTTA	115
Ngo406_3	CATCATTGGAAACCCAACAA	AGGGCTAATTCCGCTTCC	97
Ngo406_19	GATATGCAAAGCAAAGCGAA	TGCATTTCTTTCATTTGTCCA	131
Ngo406B	CCGGTTATGTTCCAAGCGAA	AAACATCACCGTTTGTTCCG	129
Ngo407	ACGCATGCAAACCGTTATGA	GCCGTTGGAAATCACGAAGA	186

1.2 Supplementary Figures

Supplementary Figure 1. Figure S1. *ngoAV* locus with location of primers used to evaluation of gene expression by qRT-PCR. Red arrows represent primers for qRT-PCR
