# Supplementary figures and images for: Phase-variable Type I methyltransferase M.NgoAV from Neisseria gonorrhoeae FA1090 regulates phasevarion expression and gonococcal phenotype
Source: Front Microbiol. 2022 Oct 4;13:917639. doi: 10.3389/fmicb.2022.917639 (PMC9577141; doi:10.3389/fmicb.2022.917639)

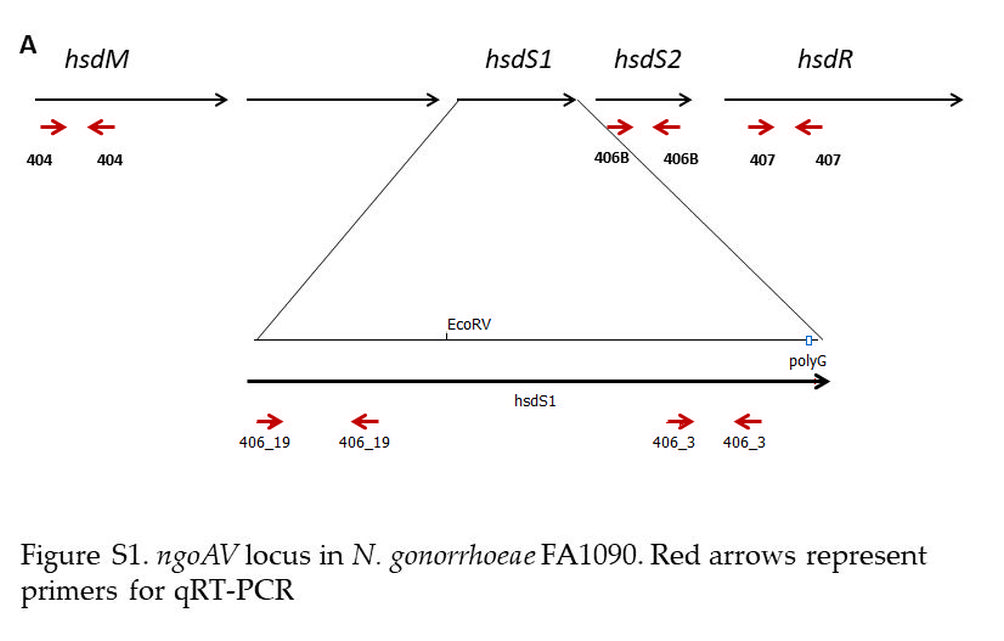

Supplement: Supplementary file 2 [file Image_1.TIF]
